# Artificial intelligence techniques for classification of Alzheimer's disease using neuroimaging data: a review

**DOI:** 10.3389/frai.2026.1748985

**Published:** 2026-05-18

**Authors:** Y. Nibila, M. Sivagami

**Affiliations:** School of Computer Science and Engineering, Vellore Institute of Technology, Chennai, India

**Keywords:** Alzheimer's disease (AD), convolutional neural network (CNN), deep learning, machine learning, magnetic resonance imaging (MRI), mild cognitive impairment (MCI), positron emission tomography (PET)

## Abstract

Alzheimer's disease (AD) is a gradually advancing brain disorder marked by memory impairment. The incurable, progressive nature of the disease leads to the dementia stage. Treatment is effective in the early stage, and it can be controlled but not cured. Artificial Intelligence **(**AI) learning models are used in medical science to detect and classify diseases into specific categories. Features are extracted from medical images and trained using AI learning models to perform an accurate diagnosis of AD. Recent advancements in machine learning (ML) and deep learning (DL) models have demonstrated significant potential in identifying AD across various data modalities, including neuroimaging, genetic information, and clinical assessments. This study focuses on the application of advanced ML and DL techniques in the identification and classification of AD, including regression models, decision trees, random forests, support vector machines (SVMs), k-nearest neighbors (KNNs), ensemble models, convolutional neural networks (CNNs), recurrent neural networks (RNNs), and generative adversarial networks (GANs). Each model is analyzed for its strengths, limitations, and performance metrics, with particular emphasis on the importance of data preprocessing and augmentation techniques to improve model accuracy and robustness. The review highlights that multimodal approaches, particularly the fusion of MRI and PET data, enhance classification accuracy compared to single-modality models. Additionally, transfer learning techniques have shown promise in overcoming data limitations by leveraging pretrained models. The review also highlights the critical role of evaluation metrics in assessing model performance, emphasizing the need for a diverse set that includes accuracy, precision, recall, F1-score, and Cohen's Kappa. The study identifies gaps in the current literature, including underreporting of certain metrics and the need for more comprehensive evaluations, and provides recommendations for future research. Finally, this study discusses the challenges and opportunities in the field, including improving model generalizability, enhancing interpretability, advanced data preprocessing and augmentation, integration with clinical workflows, and multimodal data fusion. This review provides consolidated information that may be useful for researchers, clinicians, and data scientists, offering insights into current trends, challenges, and future research directions in AI-driven AD detection.

## 1 Introduction

Alzheimer's disease (AD) is a neurodegenerative disease in elderly people, which destroys memory, slows down the ability to think, and impairs the ability to carry out simple tasks. It is caused by the buildup of two key proteins in the brain, namely amyloid beta and tau protein, which results in the death of nerve cells. The progress of AD can be classified as three stages: (i) The asymptotic stage, (ii) Mild AD, and (iii) The Dementia stage. In the first stage, changes in the brain begin to occur without any behavioral symptoms. In the second stage, memory complaints may become noticeable to patients (Mahendran and PM, 2022). In the final dementia stage, they are severely impaired and unable to manage their daily lives. If it is diagnosed at an early asymptomatic stage, treatment is effective and can control the disease but not cure it. Recent disease-modifying therapies targeting amyloid-beta plaques have shown modest slowing of cognitive decline in early stages. Lecanemab (Cohen et al., 2023) and donanemab (Courade et al., 2025) are two FDA-approved monoclonal antibodies that reduce amyloid plaque and slow down the progression by approximately 27–35% over 18 months in amyloid-positive patients with mild cognitive impairment or mild AD. But no approved therapies are available to completely stop the progression. To slow down the progression and ensure effective treatment, techniques are needed to detect and assess the disease. Artificial Intelligence (AI) has increasingly been applied in the study of neurological disorders for early diagnosis and disease monitoring. For example, Aresta et al. (2025a) proposed a digital phenotyping framework using natural language processing to analyze the linguistic patterns for Parkinson's disease detection, while the systematic review by Palmirotta et al. (2024) highlighted the diagnostic potential of linguistic markers analyzed through AI techniques. In the context of AD, Battista et al. (2020) demonstrated that AI models, when combined with neuropsychological assessments, can support early diagnosis and disease progression analysis. More recently, Aresta et al. (2025b) introduced an AI-based framework for staging and predicting the progression of individuals at risk of AD. Sorino et al. (2025) used explainable AI techniques to detect label noise in longitudinal Alzheimer's datasets, improving data reliability and model interpretability. These studies demonstrate the growing role of AI in neurological disease research and motivate its application in neuroimaging-based analysis for AD diagnosis.

There are many medical imaging modalities available to capture medical images. AD is diagnosed using this image-based approach, based on factors like size, shape, and volume. Some medical imaging modalities are X-rays, ultrasound imaging, computed tomography (CT), magnetic resonance imaging (MRI), positron emission tomography (PET), structural MRI (sMRI), and functional MRI (fMRI; Liu et al., 2020). In medical imaging, the raw image obtained from any modality may have noisy and redundant data. So the raw image is first segmented, from the segmented image, features are extracted, and then it is trained with an AI learning model to improve accuracy in classifying AD. In detecting AD, highly detailed X-rays played a major role in the past, but now they are used only for certain conditions. Ultrasound imaging lacks directional functional connectivity at a high temporal resolution. In a CT scan, mild changes in the brain cannot be detected (Bron et al., 2021). Hence, from the above imaging modalities, MRI and PET are most widely used for their image quality. Fusion of MRI and PET gives a quality image that provides more information than a single image. T1-weighted sequences, such as magnetization-prepared rapid acquisition gradient echo (MPRAGE) MRI, provide detailed structural information about the brain, whereas 18F-FDG (Fluorodeoxyglucose) PET offers insights into metabolic activity and the presence of amyloid plaques (KP and Thiyagarajan, 2022). By fusing these data sources, AI models can achieve a more comprehensive understanding of the disease, leading to more accurate and early diagnoses.

**Figure 1:**
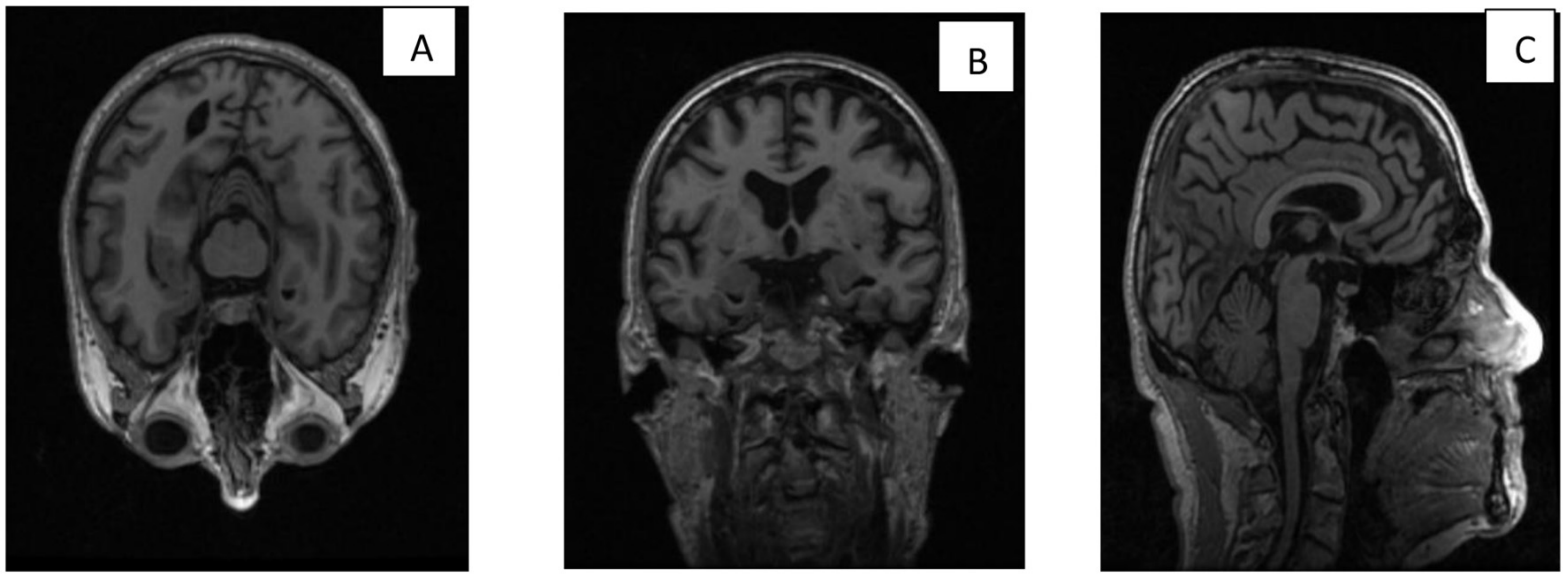
**(A)** Axial, **(B)** coronal, and **(C)** sagittal, views of brain MRI.

Figure 1 shows (A) the brain surface from top view is axial, (B) the brain surface from front or back is coronal, (C) the brain surface from side view is sagittal. In addition to imaging modalities, other biomarkers are used to detect AD. The cerebrospinal fluid (CSF) that surrounds the brain and spinal cord provides nutrition and chemicals to maintain the health of the brain cells. Analysis of cerebrospinal fluid (CSF) provides valuable diagnostic information through alterations in key protein biomarkers, such as reduced amyloid-beta 42 and increased total tau level. The presence of beta-amyloid can also be detected using blood tests, but it is less accurate than CSF (El-Sappagh et al., 2020). Genetic testing is another approach that analyzes DNA from blood or saliva, because mutations in genes will also lead to AD.

Some common AI models used for classification include support vector machine (SVM), random forest (RF), and convolutional neural network (CNN). The use of advanced AI techniques, such as CNNs and recurrent neural networks (RNNs), has enabled the automatic extraction of relevant features from neuroimaging data, reducing reliance on manual feature selection and improving the robustness of diagnostic models (Zhang et al., 2021). A transfer learning model is also used, as it takes pretrained models for image classification (Kumar et al., 2022). Despite many advancements, several challenges remain in the application of AI for AD diagnosis. These include the need for large, high-quality datasets, the complexity of integrating multimodal data, and the interpretability of AI models (Guan et al., 2021). Accuracy is an important attribute in classification. Thus, choosing a correct model based on accuracy helps to save many lives.

**Figure 2:**
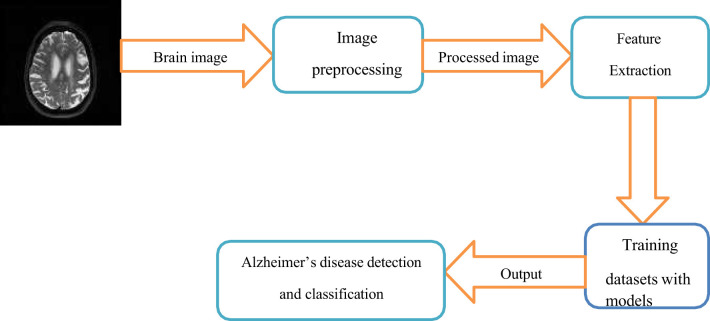
Basic steps in Alzheimer's disease classification.

Figure 2 shows that first, the Brain Image was obtained (MRI, PET, or other neuroimaging data). In preprocessing, image quality was enhanced, noise was reduced, and the images were standardized to a particular template. Relevant biomarkers such as brain volume, cortical thickness, and texture features are identified during feature extraction. Then, AI models are trained on labeled datasets, and stages of AD are predicted based on extracted features.

This review aims to provide a comprehensive overview of the recent advancements in AI techniques for the early identification and classification of AD using neuroimaging data. By examining the strengths and limitations of various ML and DL models, as well as the integration of multimodal data, this study seeks to highlight the potential of AI in transforming the diagnosis and management of AD.

The structure of the paper is described as follows:

Following the Introduction, the remainder of the paper is organized as follows. Section 2 outlines the survey methodology, including the literature search strategy and selection criteria. Section 3 reviews the most commonly used datasets (primarily ADNI and OASIS) and describes essential data preprocessing and augmentation techniques. Section 4 focuses on traditional machine learning (ML) approaches, presenting individual models (such as SVM, Random Forest, and ensemble methods), whereas Section 5 presents a comparative analysis of their performance in AD classification. Section 6 shifts to deep learning (DL) techniques, covering prominent architectures (including CNNs, RNNs/LSTMs, and GANs). Section 7 examines the evaluation metrics employed across studies, while Section 8 discusses key challenges and limitations in current approaches. Finally, Section 9 provides concluding remarks and directions for future research.

## 2 Survey methodology

This review article presents recent ML and DL techniques for AD detection. This inference is needed to identify research gaps and help to design better models to achieve accurate results. And this review is intended to help researchers learn about recent techniques in single draft to continue their research in the right way.

### 2.1 Research questions and PICO framework

This review was conducted following the methodological guidelines for systematic reviews proposed by De-La-Cruz et al. (2022). This is guided by the following three Research Questions (RQs), which structure the scope of literature, the analysis of models, and the evaluation of multimodal strategies.

RQ1 —What ML/DL techniques have been proposed for AD classification using neuroimaging data?
RQ2 —What comparative performance do these models report in terms of accuracy, precision, recall, and F1-score?
RQ3 —What data modalities and multimodal fusion strategies have demonstrated the highest classification efficacy?

The scope of the systematic review is clearly defined by the PICO framework (Petersen et al., 2015), thereby improving the quality and precision of the literature. Table 1 defines the four elements of this review.

**Table 1:** PICO framework for this review.

Element	Definition in the context of this review
Population (P)	Research studies evaluating ML or DL models for the detection or classification of AD using human subject data.
Intervention (I)	ML and DL models applied to neuroimaging or multimodal clinical data include SVM, random forest, KNN, decision trees, CNN, RNN, GAN, transformer-based models, and ensemble approaches.
Comparison (C)	Performance is evaluated across different datasets (ADNI, OASIS), modality types (unimodal vs. multimodal), and classification (binary vs. multiclass).
Outcome (O)	Reported classification performance metrics such as accuracy, precision, recall, F1-score, AUC-ROC, and Cohen's Kappa, and identified research gaps.

### 2.2 Inclusion and exclusion criteria

A systematic literature search was conducted across four major electronic databases, including PubMed, Scopus, IEEE Xplore, and Web of Science, with publications from 2018 to 2025. The search terms used were combinations of “Alzheimer's disease,” “mild cognitive impairment,” “classification,” “detection,” “diagnosis,” “ML,” “DL,” “convolutional neural network,” “MRI,” “PET,” “EEG,” “multimodal,” and “neuroimaging.” Additional articles were identified through manual screening of reference lists and Google Scholar. The total initial yield was 674 records across all sources.

Studies were included if they have (1) proposed or evaluated a ML or DL method for the classification or staging of AD or mild cognitive impairment; (2) used neuroimaging, electrophysiological, genomic, clinical, or multimodal input data from human subjects (3) were published in English in a peer-reviewed journal or conference proceedings; and (4) reported quantitative classification performance metrics such as accuracy, AUC, F1-score, or sensitivity/specificity.

Studies were excluded if they (1) were review articles, survey papers, or conference abstracts lacking a full-text experimental evaluation, (2) focused exclusively on non-classification tasks such as image segmentation, drug response prediction, or biomarker discovery without a classification outcome, or (3) provided insufficient methodological detail to assess reproducibility and did not use validated AD datasets.

### 2.3 Study selection process (PRISMA)

**Figure 3:**
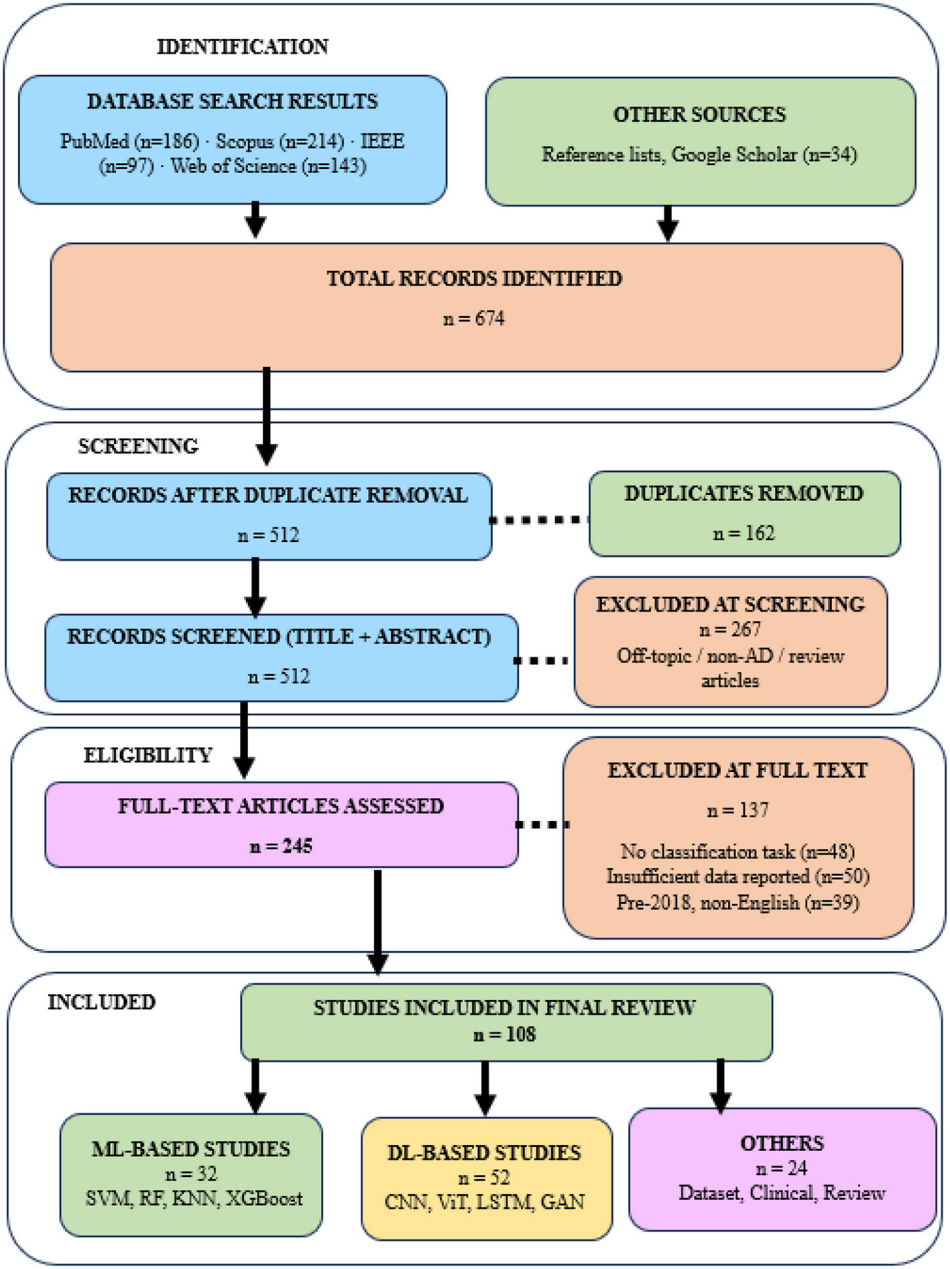
Prisma flow diagram.

After removing 162 duplicates, 512 unique records were screened by title and abstract, of which 267 were excluded as irrelevant (off-topic, non-AD, or secondary literature). The remaining 245 full-text articles were assessed for eligibility, and 137 were excluded for absence of classification task (*n* = 48), insufficient quantitative results or experimental detail (*n* = 50), and pre-2018 publication date or non-English language (*n* = 39). A final set of 108 references was included in this review, comprising 84 primary AD classification studies, 10 foundational model/algorithm references, 5 dataset references, 7 review and clinical background references, and 2 systematic review methodology references. The selection process is summarized in the PRISMA flow diagram as seen in Figure 3. The 84 primary classification studies were categorized into two groups: traditional ML methods (*n* = 32) and DL methods (*n* = 52). Multimodal and ensemble approaches are classified under their respective ML or DL category based on the primary model architecture used.

## 3 Datasets

**Figure 4:**
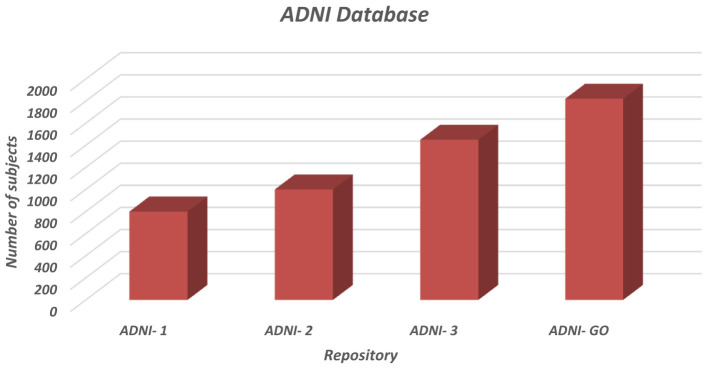
Graphical representation of the ADNI database.

Most studies have used data from the Alzheimer's Disease Neuroimaging Initiative (ADNI) and OASIS (Open Access Series of Imaging Studies). ADNI is the largest and most widely used database. The ADNI repository contains imaging, genetic, and clinical data. It includes study characteristics such as ADNI-1 (800 subjects), ADNI-2 (1,000 subjects), ADNI-3 (1,450 subjects), and ADNI-GO (1,821 subjects). Each study characteristic comprises normal control (NC), mild cognitive impairment (MCI), Early MCI, late MCI, and AD subjects. Figure 4 shows the details of the ADNI dataset in graphical form.

**Figure 5:**
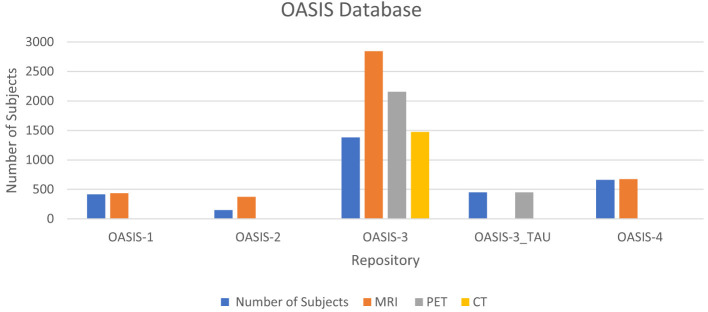
Graphical representation of the OASIS database.

OASIS have five sets: OASIS-1, OASIS-2, OASIS-3, OASIS-3_TAU, and OASIS-4. OASIS-1 is a cross-sectional collection of 416 subjects with 434 MRI sessions. A longitudinal collection of 150 participants with 373 MRI sessions is included in OASIS-2. OASIS-3 has 1,379 subjects with 2,842 MRI, 2,157 PET, and 1,472 CT sessions. OASIS-3_TAU has 451 subjects with 451 PET sessions. OASIS-4 has 663 subjects with 676 MRI sessions. Figure 5 shows the details of the OASIS dataset in graphical form.

Table 2 summarizes the most widely used datasets in the literature along with their subjects and modalities.

**Table 2:** Datasets used in literature.

Dataset	Data type	Subjects	Imaging modalities	Additional features	References
ADNI (Alzheimer's Disease Neuroimaging Initiative)	MRI, PET, genetic, CSF, clinical	1,800	Structural and functional MRI, PET	Cognitive scores, genetic data, CSF biomarkers	Weiner et al., 2013
OASIS (open Access Series of Imaging Studies)	MRI, clinical	1,379	T1-weighted MRI	Demographic & cognitive assessments	Marcus et al., 2010
NACC (National Alzheimer's Coordinating Center)	Clinical, MRI	40,000	MRI (limited subset)	Comprehensive cognitive assessments, biomarkers	Beekly et al., 2007
AIBL (Australian Imaging Biomarkers and Lifestyle)	MRI, PET	1,100	MRI, PET	Genetic information, lifestyle, and cognitive factors	Ellis et al., 2009
UK Biobank	MRI, clinical, genetic	500,000	MRI	Genetic information, lifestyle, and health records	Sudlow et al., 2015

Each of these datasets provides unique advantages for AD research.

- ADNI is the most comprehensive dataset, widely used for training and validation in AI-based AD detection.
- OASIS provides high-quality MRI images, but it has a smaller sample size.
- NACC offers one of the largest clinical datasets, but it contains only limited imaging data.
- AIBL and UK Biobank contribute multimodal data, including the genetic and lifestyle factors affecting AD progression.

### 3.1 Data preprocessing techniques

Neuroimaging data must undergo extensive preprocessing before being used in AI models. The most common preprocessing steps included in the literature are listed below.

#### 3.1.1 Data cleaning and normalization

- **Skull Stripping**: It is the process of removing the skull and other non-brain structures from brain images. In a study by Rallabandi et al. (2020), non-brain structures were removed from the image using FSL's Brain Extraction Tool.
- **Intensity Normalization**: It is adjusting the intensity values of an image to a predefined value. In a study by Raju and Gopi (2021), voxel intensity values were standardized to reduce the variability caused by scanners.
- **Motion Correction**: Blurring of images may cause by movement of patients during scans. So scans were aligned to reduce motion artifacts and improve image quality (Qu et al., 2021).
- **Spatial Normalization**: Images are registered to a standard brain atlas (MNI space) using affine (Arco et al., 2021) and non-linear registration.
- **Segmentation**: It is the process of separating brain (Arco et al., 2021) structures into different regions, such as gray matter, white matter, and CSF, for feature extraction.

#### 3.1.2 Feature extraction from imaging data

Once pre-processed, neuroimaging data undergoes feature extraction:

- **Volumetric Analysis**: In this analysis, regions of the brain affected due to Alzheimer's disease, like hippocampal volume and cortical thickness (Rallabandi et al., 2020), were extracted. This process uses tools like Freesurfer, which is used for automated subcortical segmentation, and ANTs (Advanced Normalization tools) is used for volumetric quantification.
- **Texture & Morphological Features**: In this, features like gray matter density (Bron et al., 2021), surface-based morphometry were extracted. This process uses tools like Freesurfer, which is used for surface-based morphometry and cortical thickness maps, and **SPM** (Statistical Parametric Mapping), which is used for voxel-based morphometry.
- **Functional Connectivity Measures**: In this fMRI and PET images, interactions between the brain regions were analyzed (Lei et al., 2020). This process uses tools such as the **CONN toolbox** (MATLAB-based) for functional connectivity and **SPM** for fMRI preprocessing and connectivity mapping.

### 3.2 Data augmentation

To overcome data limitations and improve AI model generalizability, augmentation techniques are important. Augmentation techniques used in this literature are listed below.

#### 3.2.1 Image-based augmentation

- **Geometric Transformations**: In this, transformations like rotation, flipping, and scaling were done (Helaly et al., 2022) to enhance diversity.
- **Intensity Transformations**: Histogram equalization, contrast adjustments (Sathiyamoorthi et al., 2021) were made to improve feature visibility.

#### 3.2.2 Synthetic data generation

- **Generative Adversarial Networks (GANs)**: Realistic MRI/PET (Sekhar et al., 2023) scans were created to address the class imbalance problem.
- **Variational Autoencoders (VAEs)**: In incomplete scans, missing brain regions (Lu et al., 2024) were generated. Table 3 describes the data preprocessing and augmentation techniques used in the studies.

**Table 3:** Analysis of data preprocessing and augmentation.

References	Model used	Data preprocessing	Data augmentation	Accuracy (%)
Sharma and Dey (2021)	Regression	Normalization, feature selection (LASSO), FDR correction, and handling missing data	Oversampling, synthetic data generation	62–92
Sudharsan and Thailambal (2021)	Decision trees	Normalization, PCA for dimensionality reduction, and handling missing data	SMOTE, oversampling	62–66
Parameswari et al. (2022)	Random forest	One-hot encoding, data imputation, and handling missing data	Oversampling, synthetic data generation	62–86
Richhariya et al. (2020)	Support vector machine	Normalization, feature scaling, recursive feature elimination (RFE), and handling missing data	Rotation, flipping, scaling	75–95
KP and Thiyagarajan (2022)	K-nearest neighbor	Normalization, feature scaling, Fisher score, and greedy search for feature selection	Oversampling	91
Qu et al. (2021)	Ensemble model	Normalization, feature selection, automated fiber quantification (AFQ), and handling missing data	Oversampling, synthetic data generation	82–98
Zhang et al. (2021)	Convolutional neural network	Skull stripping, normalization, segmentation, and feature extraction (CAM)	Rotation, flipping, scaling	76–98
Hong et al. (2019)	Recurrent neural network	Normalization, segmentation, and handling missing data	Oversampling, synthetic data generation	88–93
Zhang et al. (2024)	Generative adversarial networks	Normalization, skull stripping, and handling missing data	Synthetic data generation	75–98

## 4 Study on machine learning models for detecting Alzheimer's disease

An **ML model** is a computational model that learns patterns and relationships directly from data to perform a specific task, such as classification, regression, clustering, or prediction. Unlike traditional rule-based algorithms, ML models automatically improve their performance by adjusting internal parameters during a training phase. Machine learning algorithms are classified into three types, namely Supervised learning, Unsupervised learning, and Reinforcement learning. In the context of AD detection using neuroimaging, most studies employ supervised learning to address classification tasks (e.g., classifying Normal vs. MCI vs. AD or predicting MCI-to-AD conversion). Unsupervised learning is occasionally applied for clustering or anomaly detection, but rarely as the primary method for diagnostic classification. This section analyzes various ML models along with the types of medical images used for AD detection and classification.

### 4.1 Regression

Regression is used to find a relationship between the dependent variable (target) and one or more independent variables (predictors). It was first termed by Francis Galton in the nineteenth century (Galton, 1885). The mathematical representation of regression is illustrated in Equation 1.


$$\begin{array}{l} \text{Y}=\text{aX}+\text{b} \end{array}$$ (1)

Where Y—dependent variable,

X—independent variable,

a and b—linear coefficients.

**Figure 6:**
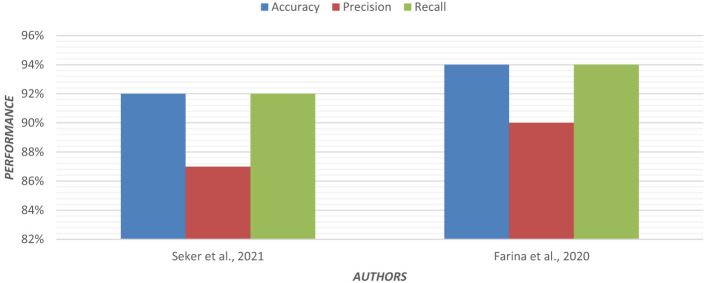
Graphical representation of the performance of the regression model.

Multinomial logistic regression was applied using Permutation Entropy (PE) for early detection of AD. In both eyes-open and eyes-closed states, EEG data from 255 subjects were analyzed. In EEG recordings, the removal of artifacts is crucial. For each EEG, epoch PE values were calculated from frontal, central, parietal, temporal, and occipital regions of the brain (Seker et al., 2021). Visualization of the PE value distribution was to discriminate between groups. In the entropy feature sets, the multinomial logistic regression model achieved higher classification accuracy. Figure 6 shows the graphical representation of the performance of the regression model.

EEG is commonly used for its low cost and portability. For comparing resting state EEG and structural MRI, both datasets were preprocessed, and measures such as MMSE, Verbal fluency, Digit span backward, and Boston Naming test were considered (Farina et al., 2020). An elastic net model, which is an extension of linear regression, was used. Structural MRI performance was better than EEG. Longitudinal tracking of EEG features is needed for better classification. Table 4 describes the contributions of the regression model used in the classification of Alzheimer's disease.

**Table 4:** Review of existing research on the regression model.

References	Main research and contributions
Şeker et al. (2021)	This proposed research calculates permutation entropy values for different regions of the brain, and a multinomial logistic regression model is used for classification.
Farina et al. (2020)	This proposed research uses structural MRI images and an extended version of linear regression (elastic net model) for classification.

### 4.2 Decision tree

**Figure 7:**
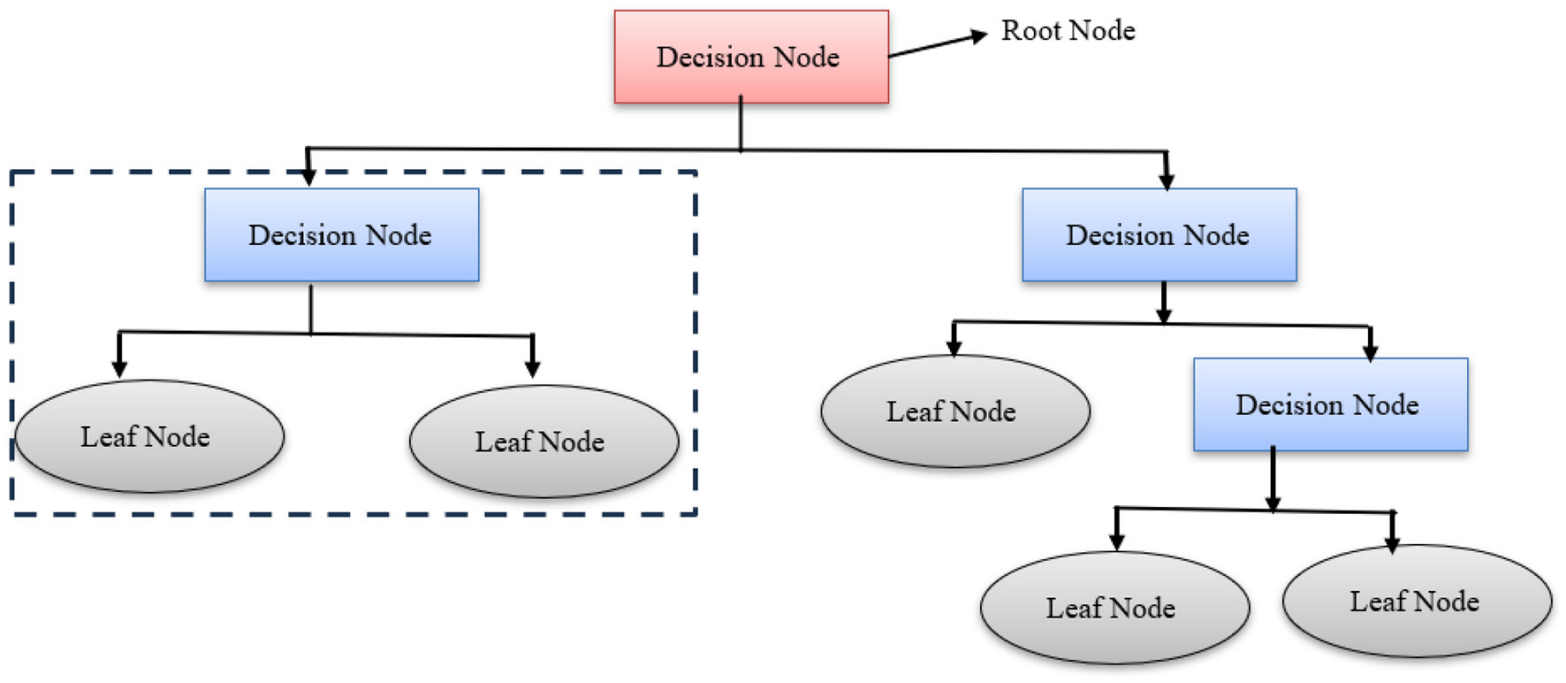
Graphical representation of a decision tree.

A decision tree is a powerful supervised learning algorithm, as termed by Quinlan (1986) was used for both regression and classification tasks. It is a tree-structured classifier with decision nodes and leaf nodes. Below is the graphical representation of a decision tree. Figure 7 represents the basic structure of decision trees used in classification. The decision tree data format is illustrated in Equation 2.


$$\begin{array}{l} \text{Data comes in records of the form }\left(\text{x},\text{Y}\right)=\left({\text{x}}_{1,}{\text{x}}_{2,}{\text{x}}_{3,\ldots \ldots ,}{\text{x}}_{\text{k},}\text{Y}\right) \end{array}$$ (2)

**Figure 8:**
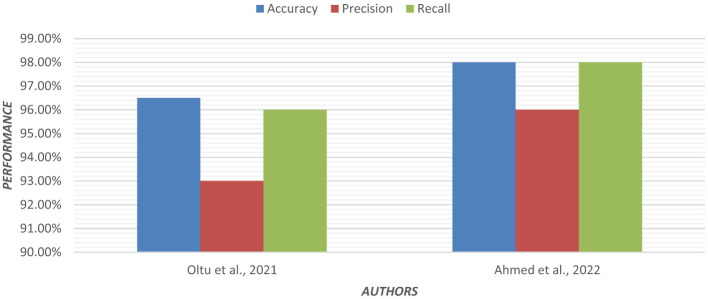
Graphical representation of the performance of the decision tree.

Where Y is the target variable to understand, classify, or generalize, and x is a vector composed of x_1_, x_2_, and x_3_ features. Figure 8 shows the graphical representation of the performance of the decision tree.

Oltu et al. (2021) extracted discriminative features from resting-state EEG signals, including sub-band power spectral density (PSD) obtained through discrete wavelet transform (DWT) and inter-hemispheric coherence measures. These features were then fed into a bagged trees classifier. This ensemble method combines multiple decision trees trained on bootstrapped subsets of the data (bagging) to improve stability and reduce variance. Decision tree ensembles were particularly valuable here because they provided interpretable rules like thresholds on specific frequency band powers or coherence values, and gave robust classification performance on noisy, non-stationary EEG data.

Ahmed et al. (2022) applied Boruta feature selection to identify the most informative single-nucleotide polymorphisms (SNPs) and integrated them with neuroimaging-derived features. Then it is trained using a Gradient Boosting Tree (GBT) model (a sequential ensemble of decision trees where each new tree corrects the errors of the previous ones). GBT was fine-tuned to classify subjects into Normal Control (NC), Mild Cognitive Impairment (MCI), and AD. The strength of the method lies in its ability to handle high-dimensional genetic data, capture non-linear interactions between SNPs and imaging features, and provide feature importance rankings. Table 5 describes the contributions of the decision tree model used in the classification of Alzheimer's Disease.

**Table 5:** Review of existing research on the decision tree.

References	Main research and contributions
Oltu et al. (2021)	EEG sub-bands are obtained using the discrete wavelet transform (DWT), and extracted features are trained using a bagged trees classifier.
Ahmed et al. (2022)	Single nucleotide polymorphisms (SNPs) serve as crucial biomarkers for predicting Alzheimer's disease (AD) and enhancing early detection through genetic analysis.

### 4.3 Random forest

Random forest is a popular supervised learning algorithm, originated in 1995 by Tin Kam Ho for both regression and classification tasks (Ho, 1995). Multiple classifiers are combined (Ensemble learning) for the better performance of the model. Mathematical representation of Random Forest is illustrated in Equation 3.


$$\begin{array}{l} \hat{y}=\frac{1}{N}\sum_{i=1}^{N}f_{i}\left(x\right) \end{array}$$ (3)

Where y is the predicted output, N is the total number of trees in the forest, and f_i_(x) is the prediction of the ith tree.

Random Forest performs well in AD classification due to its robustness to overfitting, ability to handle high-dimensional and heterogeneous data, and built-in feature importance ranking, which helps in biomarker interpretation.

**Figure 9:**
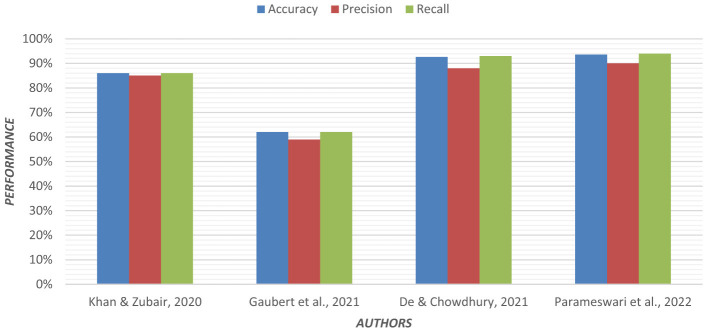
Graphical representation of the performance of random forest.

Khan et al. (2022) developed a multimodal ML pipeline using longitudinal MRI images from the OASIS database. After preprocessing and feature extraction, Random Forest was applied among multiple classifiers and achieved the highest accuracy for early AD detection. Its robustness to multimodal features and ability to handle class imbalance contributed to reliable classification. Gaubert et al. (2021) combined multimodal biomarkers (amyloid status, neurodegeneration, cognitive scores) to screen for preclinical AD. Random Forest was selected as the best classifier for its ability to handle high-dimensional, correlated data and to provide feature importance, highlighting key predictors of amyloid and cognitive impairment. Figure 9 shows the graphical representation of the performance of Random Forest.

De and Chowdhury (2021) proposed a rank-modulated fusion approach on Diffusion Tensor Imaging (DTI) data. CNNs and Random Forest were trained separately on features like Fractional Anisotropy (FA), Mean Diffusivity (MD), and Echo Planar Imaging (EPI). At the decision level, Random Forest predictions were fused with CNN outputs using a modulated ranking strategy, enabling accurate multiclass classification (CN, AD, MCI). The ensemble nature of Random Forest helps to improve stability with deep features. Parameswari et al. (2022) addressed missing and irregularly sampled data in thermal analysis of AD phases. Random Forest was employed after one-hot encoding and imputation, because of its tolerance for noisy/incomplete inputs and ability to rank features for phase categorization.

Across these studies, Random Forest consistently performs well on diverse data types, including OASIS (longitudinal MRI), multimodal clinical or neurodegeneration data, DTI, and thermal data with extracted features such as MRI or DTI metrics, cognitive scores, amyloid or neurodegeneration markers, and thermal signatures. Evaluation focused on accuracy ranging from 85 to 95%, in addition to robustness to high-dimensional data. The ensemble nature of Random Forest provides superior generalization and stability compared to single classifiers, making it suitable for early detection. Table 6 describes the contributions of the Random Forest model used in the classification of Alzheimer's Disease.

**Table 6:** Review of existing research on random forest.

References	Main research and contributions
Khan et al. (2022)	The multimodal machine learning (ML) approach significantly aids in the early detection of Alzheimer's disease (AD) through a structured five-stage pipeline: preprocessing, segregation, modeling, model prediction, and performance metrics.
Gaubert et al. (2021)	The key objective of this study is to determine the amyloid status, neurodegeneration status, and cognitive impairment level of the patient.
De and Chowdhury (2021)	A rank-modulated fusion of convolutional neural networks (CNNs) and random forest classifiers, trained on diffusion tensor imaging (DTI) features like FA, MD, and EPI, enables accurate classification of Alzheimer's disease (AD) and its stages.
Parameswari et al. (2022)	Random forest classifiers are used to handle missing and irregularly sampled data in Alzheimer's disease studies, employing techniques like one-hot encoding and data imputation.

### 4.4 K nearest neighbor

The KNN algorithm is a basic classification algorithm developed by Fix and Hodges (1951). It is widely used in pattern recognition, data mining, and intrusion detection. In KNN, distance metrics are used to identify the nearest or closest points in a dataset. Mathematical equation of KNN is illustrated in Equation 4.


$$\begin{array}{l} \text{Euclidean distance }(\text{x},{\text{X}}_{\text{i}})=\sqrt{\sum_{j=1}^{d}(x_{j}-\;X_{ij\;}}{)}^{2} \end{array}$$ (4)

In AD studies, KNN has been applied both independently and with feature selection or optimization techniques to handle high-dimensional neuroimaging or multimodal data. KP and Thiyagarajan (2022) combined Fisher Score with greedy search for efficient feature selection, then applied KNN on multimodal data (MRI and clinical features). KNN achieved strong classification performance by reducing dimensionality without losing discriminative information.

**Figure 10:**
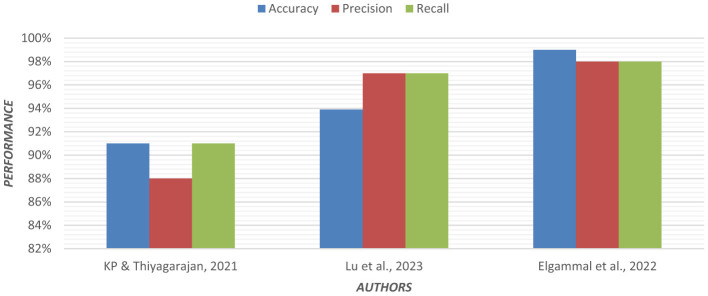
Graphical representation of the performance of K nearest neighbor.

Lu et al. (2023) optimized a fuzzy KNN variant using the salp swarm algorithm to improve classification accuracy on neuroimaging-derived features. The flexible nature of KNN, paired with metaheuristic tuning, provides better classification in AD stages. Elgammal et al. (2022) proposed a multifractal geometry-based strategy to enhance feature representation before applying KNN. This enables early-stage AD detection by capturing complex structural patterns in MRI data. Figure 10 shows the graphical representation of the performance of K-Nearest Neighbor.

These studies show KNN's effectiveness when combined with careful feature engineering or optimization, particularly in small or medium datasets. However, KNN's performance can degrade in very high-dimensional raw images without dimensionality reduction. Table 7 describes the contributions of the K Nearest Neighbor model used in the classification of Alzheimer's Disease.

**Table 7:** Review of existing research on K-nearest neighbor.

References	Main research and contributions
KP and Thiyagarajan (2022)	A fusion-based algorithm combining greedy search and Fisher score effectively selects features from multimodal data for Alzheimer's disease classification, evaluated on 2,737 subjects using LOOCV and 10-fold cross-validation with KNN.
Lu et al. (2023)	A fuzzy K-nearest neighbor method based on the improved binary salp swarm algorithm (IBSSA-FKNN) is proposed for the early diagnosis of AD.
Elgammal et al. (2022)	This study proposed a multifractal geometry-based feature extraction method combined with K-nearest neighbor (KNN) classification for early Alzheimer's disease (AD) detection.

### 4.5 Support vector machine

Support Vector Machine is a supervised ML algorithm for linear or non-linear regression and outlier detection tasks. It is adaptable and can manage high-dimensional data. It was developed by Vladimir Vapnik at AT&T Bell Laboratories (Cortes and Vapnik, 1995). Mathematical representation of SVM is illustrated in Equation 6.


$$\begin{array}{l} \text{f}\left(\text{x}\right)={\text{w}}^{\wedge }{\text{T}}^{*}\text{x}+\text{b} \end{array}$$ (5)

Where f(x) is the function that classifies the input x, w denotes weight vector, x denotes input vector and b denotes bias term.

Multivariate pattern analysis using a searchlight approach was applied to integrate MRI and neuropsychological data. Searchlight features produced more informative representations than traditional dimensionality reduction techniques such as Principal Component Analysis (PCA), leading to improved SVM classification performance (Arco et al., 2021). Similarly, studies using longitudinal MRI datasets combined with cognitive measures such as the Mini Mental State Examination (MMSE), Clinical Dementia Rating (CDR), estimated Total Intracranial Volume (eTIV), normalized Whole Brain Volume (nWBV), and Atlas Scaling Factor (ASF) reported that SVM with a linear kernel outperformed other ML algorithms including Decision Tree, Random Forest, Naïve Bayes, and Logistic Regression (Kishore et al., 2021).

**Figure 11:**
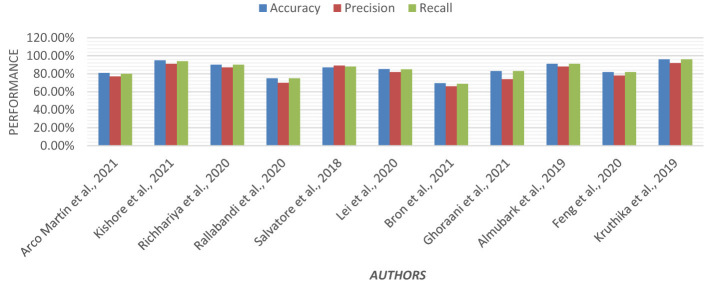
Graphical representation of the performance of SVM.

Feature selection is another important factor influencing SVM performance in AD classification. Methods such as Universum Support Vector Machine–Recursive Feature Elimination (USVM-RFE) have been used to identify discriminative brain tissue features, including gray matter, white matter, and cerebrospinal fluid. This enables improved classification accuracy by reducing redundant features (Richhariya et al., 2020). In large neuroimaging datasets, cortical thickness features extracted from structural MRI scans of 1,167 subjects from the ADNI database were used to train various ML models. In that study, non-linear SVM achieved the highest classification accuracy for distinguishing MCI and AD subjects (Rallabandi et al., 2020). Furthermore, the study by Salvatore et al. (2018) demonstrated that MRI-based ML approaches can characterize the progressive course of AD and predict conversion from mild cognitive impairment to Alzheimer's dementia up to 24 months before clinical diagnosis. It highlights the importance of MRI biomarkers for early detection. Figure 11 shows the graphical representation of the performance of SVM.

**Figure 12:**
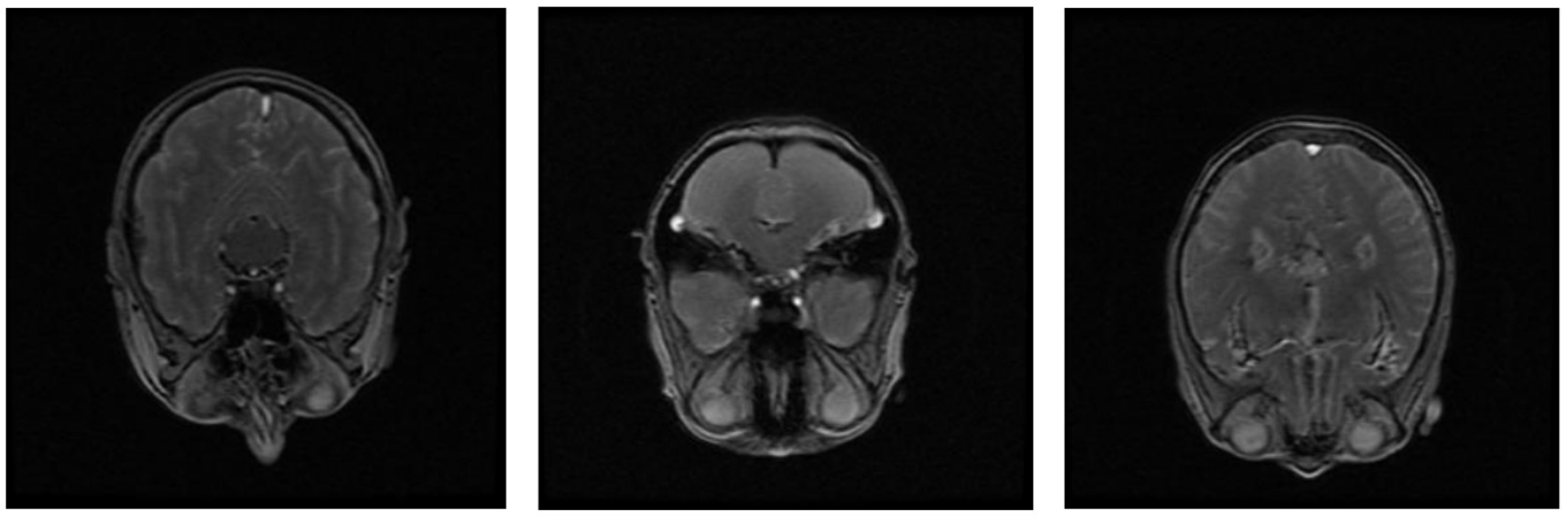
Sample brain MRI of CN, MCI, and AD.

Several studies have also explored multimodal neuroimaging approaches to enhance SVM performance. Functional and structural brain data derived from fMRI and diffusion tensor imaging (DTI) have been used to construct brain connectivity networks, where SVM classifiers effectively utilized these features for AD prediction (Lei et al., 2020). In other words, gray matter density features were encoded into modulated gray matter maps after preprocessing structural MRI data. In this SVM with a linear kernel, strong classification accuracy was achieved using cross-validation strategies (Bron et al., 2021). Figure 12 shows the MRI images of Cognitively Normal, Mild Cognitive Impairment, and AD subjects.

Beyond neuroimaging data, SVM has also been applied to alternative biomarkers and behavioral assessments. Gait analysis combined with Montreal Cognitive Assessment (MoCA) scores was used to detect mild cognitive impairment (MCI). SVM models trained on gait features demonstrated promising results for early detection of cognitive decline (Ghoraani et al., 2021). Similarly, cognitive and neuropsychological datasets evaluated using cross-validation approaches showed that SVM outperformed other ML models such as Random Forest, Gradient Boosting, and AdaBoost in predicting AD (Almubark et al., 2019). Table 8 describes the contributions of SVM used for the classification of Alzheimer's disease.

**Table 8:** Review of existing research on the SVM model.

References	Main research and contributions
Kishore et al. (2021)	A longitudinal study of 150 subjects used MRI data and features like MMSE, CDR, eTIV, nWBV, and ASF to evaluate cognitive impairment and brain volume. Among five machine learning models, the support vector machine (SVM) with a linear kernel and C = 2 achieved the best performance.
Arco et al. (2021)	Searchlight analysis, a multivariate pattern method, fuses MRI and neuropsychological data to extract rich features, with SVM classifiers outperforming PCA in predicting Alzheimer's disease.
Ghoraani et al. (2021)	Mild cognitive impairment (MCI) detection can be enhanced through gait assessments and machine learning, using 78 community-dwelling older adults who underwent the Montreal cognitive assessment (MoCA) alongside gait evaluations.
Richhariya et al. (2020)	Universum support vector machine-based recursive feature elimination (USVM-RFE) is an effective technique for selecting discriminative features from structural MRI data, including gray matter, white matter, and cerebrospinal fluid, to accurately diagnose Alzheimer's disease.
Rallabandi et al. (2020)	Structural MRI analysis from the ADNI database involved preprocessing steps like skull stripping and tissue segmentation, utilizing 68 features related to cortical thickness for automatic classification of Alzheimer's disease (AD). A non-linear support vector machine (SVM) achieved the highest accuracy.
Feng et al. (2020)	Wavelet transformation energy features (WTEF) derived from preprocessed gray matter images in structural MRI are used to detect Alzheimer's disease, with the combined energy features at different directions and transformation levels classified using a nearest neighbor (NN) classifier.
Lei et al. (2020)	Multimodal data from functional MRI (fMRI) and diffusion tensor imaging (DTI) undergo preprocessing using automatic anatomical landmark (AAL) techniques. Features are extracted from both functional and structural inputs through non-convex multitask learning, and support vector machine (SVM) classifiers are utilized for the final classification of the data.
Kruthika et al. (2019)	A multistage classifier combining KNN, SVM, and Naive Bayes effectively categorizes Alzheimer's disease stages by selecting features using particle swarm optimization (PSO).

Additional feature extraction methods include Wavelet Transformation Energy Features (WTEF) derived from structural MRI gray matter images (Feng et al., 2020), as well as multistage classification frameworks combining algorithms such as KNN, Naïve Bayes, and SVM with Particle Swarm Optimization for improved feature selection and stage-wise AD diagnosis (Kruthika et al., 2019). Overall, these studies demonstrate that the effectiveness of SVM in AD classification is strongly influenced by feature extraction strategies, kernel selection, and multimodal data integration.

### 4.6 Extreme learning machine approaches

**Figure 13:**
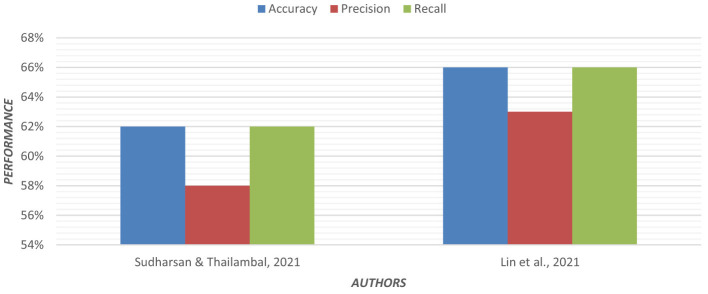
Graphical representation of the performance of the ELM model.

A Regularized Extreme Learning Machine (RELM) based decision tree was used to train 214 subjects from ADNI. Cross-validation is performed to check permutation, whereas Principal Component Analysis (PCA) is utilized for K-fold and leave one out (LOO) were the two cross validation technique used in this system (Sudharsan and Thailambal, 2021). The classifiers used were Support Vector Machine (SVM), Regularized Extreme Learning Machine (RELM), and Import Vector Machine (IVM). Among the three classifiers, RELM achieves the highest accuracy. The proposed method can be used with some other image modalities. Figure 13 shows the graphical representation of the performance of the ELM model in AD classification.

Fusion is a major challenge in the use of multimodal data. In AD diagnosis, images were fused using the Linear Discriminant Analysis (LDA) score method (Lin et al., 2021). The four multimodalities were MRI, Positron Emission Tomography (PET), cerebrospinal fluid biomarkers, and genetic features. In the data preprocessing, age correction, feature selection, and reduction were performed. After preprocessing, the data were individually scored using LDA. The progress of AD in different modalities was represented using this score. The scores were concatenated and finally an Extreme Learning Machine (ELM) based Decision Tree performs the classification based on the scores. This Decision tree has better performance. For fusion of multimodal data efficient method can be used. Table 9 describes the contributions of the ELM model in AD classification.

**Table 9:** Review of existing research on ELM.

References	Main research and contributions
De and Chowdhury (2021)	In this, a regularized extreme learning machine (RELM) based decision tree was used, and cross-validation was performed to check the permutation.
Lin et al. (2021)	In this study, images are fused using the linear discriminant analysis (LDA) score method, and an extreme learning machine (ELM) based decision tree performs the classification based on the scores.

### 4.7 Ensemble model

Ensemble learning is a ML algorithm that combines the predictions of multiple individual models for better performance than a single model. It is an alternative to a weak learning algorithm. The first ensemble algorithm was introduced by Freund and Schapire (1997). Mathematical representation of Ensemble model is illustrated in Equation 6.


$$\begin{array}{l} \text{f(x)}=\;\sum_{i=1}^{N}w_{i\;}h_{i}\left(x\right)\; \end{array}$$ (6)

Where f(x) is prediction for input x, N is the number of base models, h_i_(x) is the prediction made by ith base model and w_i_ is the weight assigned to the ith base model. Table 10 describes the contributions of the Ensemble model used in the classification of Alzheimer's disease.

**Table 10:** Review of existing research on the ensemble model.

References	Main research and contributions
Qu et al. (2021)	Automated fiber quantification (AFQ) is utilized to extract diffusion measurements along 18 white matter fiber tracts, employing a deterministic streamlines tracking algorithm to trace whole brain fibers, clean abnormal fibers, and resample each fiber for accurate analysis.
Ryu et al. (2020)	This approach, which includes the extraction of derived variables and parameter optimization, has shown improved accuracy.
Khan et al. (2022)	XGB handles complex patterns, DT offers interpretability, and SVM excels in high-dimensional spaces. With these strengths, the ensemble model outperformed individual models.
Ma et al. (2023)	The ensemble model integrates a three-dimensional residual convolution module with a broad learning system (BLS) to capture deep features from MRI images. This ensemble outperformed previous models like 3D-ResNet and VoxCNN.
Chen et al. (2023)	Using multiple convolutional neural networks (CNNs) such as ResNet50, DenseNet121, and EfficientNet_B0 with transformer architectures demonstrated superior performance than individual models.
Jenber Belay et al. (2024)	A combination of deep ensemble learning techniques with quantum machine learning approaches was used. This hybrid method has better results.
Kavitha et al. (2022)	Different machine learning algorithms, including decision tree classifier, random forest classifier, support vector machine (SVM), and XGBoost, were trained and then ensembled to predict the final result.
Sharma et al. (2022)	This study proposed an ensemble of a regularized regression model and a random forest. To support the early detection of AD, gene data is tested.

Diffusion Tensor Imaging (DTI) captured white matter and served as a biomarker for AD (Qu et al., 2021). In this, DICOM images were converted to Nifti format. Preprocessing involved head motion correction, skull stripping, etc. Automated Fiber Quantification (AFQ) was used to extract diffusion measurements along with 18 white matter fiber tract features. In AFQ, deterministic streamlines tracking algorithm was used to track whole brain fiber, abnormal fibers were cleaned, resampling was done to each fiber. The extracted features were trained using XGBoost for classification. It can be used for a large dataset (Ryu et al., 2020). Extractions of derived variables and parameter optimization using the XGBoost classifier had better accuracy.

Extreme Gradient Boosting (XGB), Decision Trees (DT), and Support Vector Machines (SVM) were used with a polynomial kernel to enhance diagnostic accuracy (Khan et al., 2022). Each model contributes its strengths: XGB handles complex patterns, DT offers interpretability, and SVM excels in high-dimensional spaces. The ensemble model achieved an accuracy of 89.77% in classifying AD, mild cognitive impairment (MCI), and cognitively normal (CN) subjects. After hyperparameter optimization using grid search, the accuracy improved to 95.75%, outperforming individual models.

**Figure 14:**
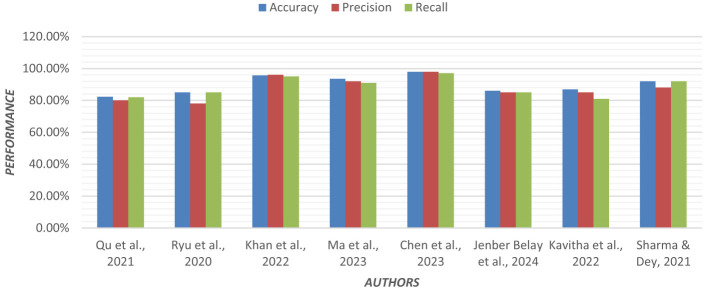
Graphical representation of the performance of the ensemble model.

The ensemble model integrates a three-dimensional residual convolution module with a Broad Learning System (BLS) to capture both deep and broad features from MRI images (Ma et al., 2023). The deep component extracts hierarchical features, while the BLS captures extensive feature interactions. MRI images from ADNI dataset were used, this ensemble outperformed previous models like 3D-ResNet and VoxCNN in accuracy, sensitivity, specificity, and F1 score. Another study combines multiple CNNs such as ResNet50, DenseNet121, and EfficientNet_B0 with Transformer architectures (Chen et al., 2023). The ensemble leverages the spatial feature extraction capabilities of CNNs and the sequence modeling strength of Transformers. The ensemble demonstrated superior performance in predicting AD and its progression compared to individual models. Figure 14 shows the graphical representation of the performance of the Ensemble model.

This innovative model merges deep ensemble learning techniques with quantum ML approaches (Jenber Belay et al., 2024). The ensemble consists of multiple DL models whose outputs were integrated and further processed using quantum algorithms to enhance pattern recognition capabilities. This hybrid approach has shown promising results in improving the accuracy and efficiency of AD diagnosis. The study employed the OASIS dataset, which includes demographic details and clinical parameters such as age, gender, education level, socioeconomic status, Mini-Mental State Examination (MMSE) scores, Clinical Dementia Rating (CDR), and Atlas Scaling Factor (ASF). Different ML algorithms, including Decision Tree Classifier, Random Forest Classifier, Support Vector Machine (SVM), and XGBoost, were trained and then ensembled to predict the final result (Kavitha et al., 2022).

**Figure 15:**
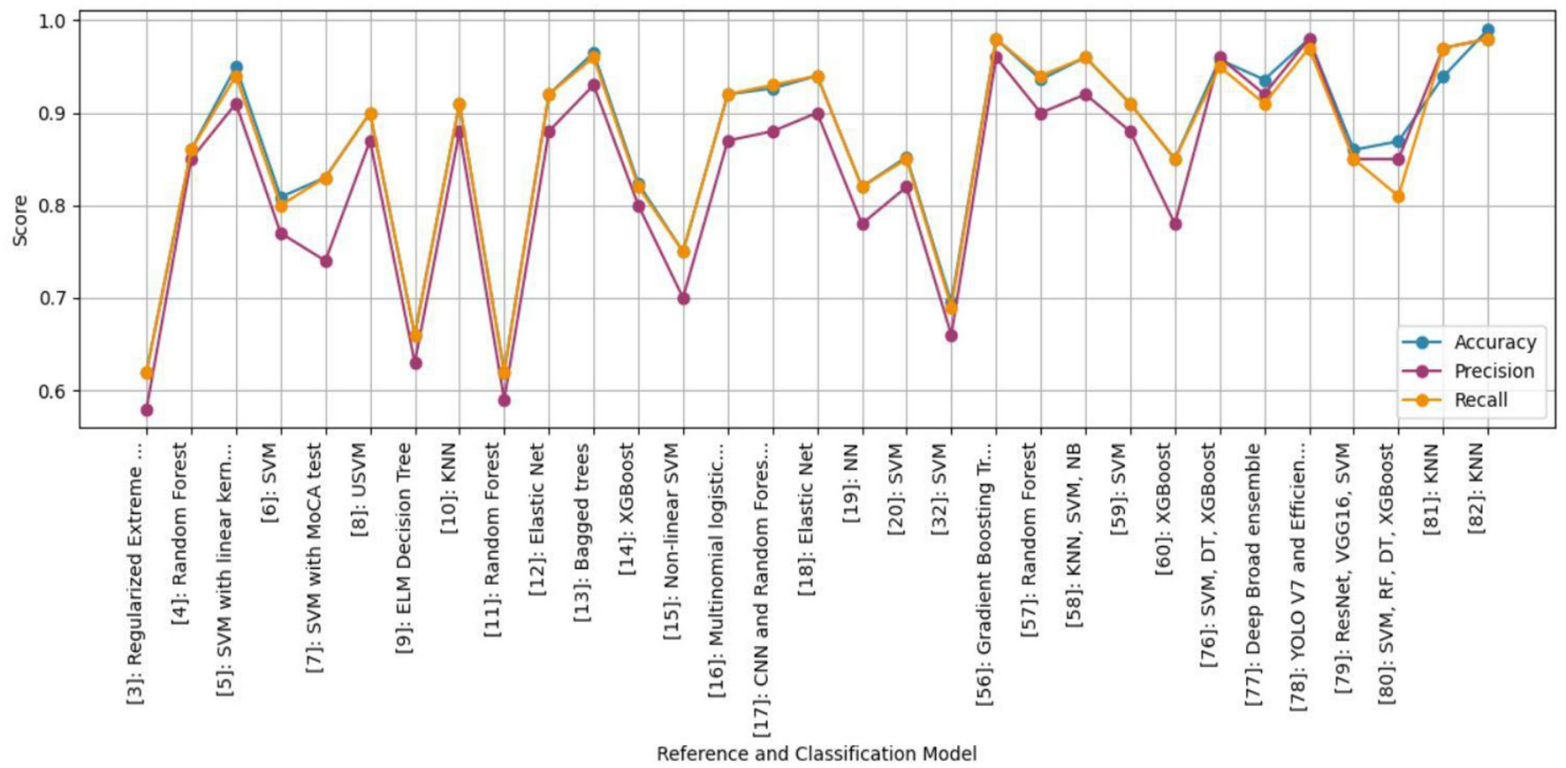
Graphical representation of the performance of machine learning models.

Using an ensemble of Random Forest and a regularized regression model (LASSO), the genetic biomarkers in the prefrontal cortex, middle temporal gyrus, hippocampus, and entorhinal cortex were explored (Sharma and Dey, 2021). Gene expression data were preprocessed from these brain regions. *P*-value was calculated, and due to a large sample size, FDR (False Discovery Rate) correction was made. Features were selected from preprocessed data by wrapping LASSO and Variable Selection using Random Forest (VarSelRF). The selected gene candidates were assessed through Elastic Net, along with Random Forest and SVM classifiers. But the maximum accuracy was achieved by the Elastic Net classifier for the middle temporal gyrus region. Gene data can be tested to support the early detection of AD. Table 11 presents the performance of different ML models based on accuracy, precision, and recall used in the literature. Figure 15 shows the graphical representation of the performance of different ML Models.

**Table 11:** Performance of different ML models.

References	Medical image	Classification model	Datasets	Comparison performed	Accuracy (%)	Precision	Recall
Sudharsan and Thailambal (2021)	sMRI	Regularized extreme learning machine (RELM)	214 subjects from ADNI	Multiclass (CN vs. MCI vs. AD)	62	0.58	0.62
Khan and Zubair (2022)	MRI	Random forest	150 subjects from OASIS	Multiclass (CN vs. MCI vs. AD)	86	0.85	0.86
Kishore et al. (2021)	MRI	SVM with linear kernel	150 subjects	MCI vs. CN	95	0.91	0.94
Arco et al. (2021)	MRI	SVM	134 subjects from ADNI	Multiclass (CN vs. MCI vs. AD)	80.9	0.77	0.80
Ghoraani et al. (2021)	Cognitive data	SVM with the MoCA test	78 subjects	MCI vs. CN	83	0.74	0.83
Richhariya et al. (2020)	sMRI	USVM	150 subjects from ADNI	AD vs. CN	90	0.87	0.90
Lin et al. (2021)	MRI, PET	ELM decision tree	746 subjects from ADNI	AD vs. MCI	66	0.63	0.66
KP and Thiyagarajan (2022)	MRI, PET, and cognitive data	KNN	1,737 from ADNI, 1,000 from AIBL	Multiclass (CN vs. MCI vs. AD)	91	0.88	0.91
Gaubert et al. (2021)	PET, MRI, EEG	Random forest	304 subjects	AD vs. CN	62	0.59	0.62
Sharma and Dey (2021)	MRI	Elastic net	200 subjects	AD vs. CN	92	0.88	0.92
Oltu et al. (2021)	EEG	Bagged trees	35 subjects	AD vs. CN	96.5	0.93	0.96
Qu et al. (2021)	DTI	XGBoost	862 subjects	AD vs. CN	82.35	0.80	0.82
Rallabandi et al. (2020)	MRI	Non-linear SVM	1,167 subjects from ADNI	MCI vs. AD	75	0.70	0.75
Şeker et al. (2021)	EEG	Multinomial logistic regression	255 subjects	AD vs. CN	92	0.87	0.92
De and Chowdhury (2021)	DTI	CNN and random forest	655 subjects	AD vs. CN	92.65	0.88	0.93
Farina et al. (2020)	sMRI	Elastic net	450 subjects	AD vs. CN	94	0.90	0.94
Feng et al. (2020)	sMRI	NN	480 subjects	AD vs. CN	82	0.78	0.82
Lei et al. (2020)	fMRI, DTI	SVM	170 subjects	AD vs. CN	85.23	0.82	0.85
Bron et al. (2021)	sMRI	SVM	1,715 subjects from ADNI	AD vs. CN	69.5	0.66	0.69
Ahmed et al. (2022)	MRI	Gradient boosting tree	757 subjects from ADNI	Multiclass (CN vs. MCI vs. AD)	98	0.96	0.98
Parameswari et al. (2022)	MRI	Random forest	416 subjects from OASIS	AD vs. CN	93.6	0.90	0.94
Kruthika et al. (2019)	MRI	KNN, SVM, NB	475 subjects from ADNI	Multiclass (CN vs. MCI vs. AD)	96	0.92	0.96
Almubark et al. (2019)	Cognitive data	SVM	156 subjects from ADNI	AD vs. CN	91	0.88	0.91
Ryu et al. (2020)	MRI	XGBoost	566 subjects from OASIS	AD vs. CN	85	0.78	0.85
Khan et al. (2022)	MRI	SVM, DT, XGBoost	2,125 images from ADNI	Multiclass (CN vs. MCI vs. AD)	95.75	0.96	0.95
Ma et al. (2023)	MRI	Deep broad ensemble	434 subjects from ADNI	Multiclass (CN vs. MCI vs. AD)	93.58	0.92	0.91
Chen et al. (2023)	MRI	YOLO V7 and Efficient net B3	461 subjects from OASIS	AD vs. CN	98	0.98	0.97
Jenber Belay et al. (2024)	MRI	ResNet, VGG16, SVM	ADNI	AD vs. CN	86	0.85	0.85
Kavitha et al. (2022)	MRI	SVM, RF, DT, XGBoost	OASIS	AD vs. CN	86.92	0.85	0.81
Lu et al. (2023)	MRI	KNN	202 subjects from ADNI	Multiclass (CN vs. MCI vs. AD)	93.9	0.97	0.97
Elgammal et al. (2022)	MRI	KNN	750 subjects from ADNI	Multiclass (CN vs. MCI vs. AD)	99	0.98	0.98
Salvatore et al. (2018)	MRI	SVM	540 subjects from ADNI	MCI vs. AD	87	0.89	0.88

## 5 Comparative analysis of machine learning models

This section provides a detailed summary of the reviewed studies and offers a comparative analysis of the performance of different ML models in AD diagnosis. The goal is to highlight the strengths, limitations, and potential of these models in improving the accuracy and reliability of AD diagnosis.

**Regression Models**:

- Regression models, particularly those combining Random Forest and regularized regression techniques like LASSO, have been effective in identifying genetic biomarkers associated with AD. For instance, researchers (Sharma and Dey, 2021) used an ensemble of Random Forest and LASSO to explore gene expression data, achieving high accuracy in identifying AD-related genes in specific brain regions.
- Multinomial logistic regression models, combined with Permutation Entropy (PE) from EEG data, have also shown promise in early AD detection, with accuracies reaching up to 92% (Seker et al., 2021). These models are particularly useful for analyzing EEG data, which is cost-effective and portable, making it suitable for large-scale screening.

**Decision Trees**:

- Decision tree-based models, such as Regularized Extreme Learning Machine (RELM) and Extreme Learning Machine (ELM), have been used for AD classification with moderate success. For example, a study (Sudharsan and Thailambal, 2021) achieved 62% accuracy using RELM on MRI data from the ADNI database.
- Decision trees have also been employed in multimodal data fusion, where Linear Discriminant Analysis (LDA) scores from MRI, PET, and genetic data were used to classify AD stages, achieving 66% accuracy (Lin et al., 2021). These models are interpretable and effective for combining data from multiple sources, but they may struggle with high-dimensional data.

**Random Forest**:

- Random Forest models have demonstrated good performance in AD classification, particularly when applied to multimodal data. In a study (Khan and Zubair, 2022) used a five-stage ML pipeline with Random Forest, achieving 86% accuracy on longitudinal MRI data from the OASIS database.
- Random Forest has also been effective in handling missing data and irregularly sampled data, making it a robust choice for AD diagnosis (Lin et al., 2021). Its ability to handle large datasets and its robustness to overfitting make it a popular choice for AD classification tasks.

**Support Vector Machines (SVM)**:

- SVM models, especially those using linear kernels, have consistently achieved high accuracy in AD classification. The study (Kishore et al., 2021) reported 95% accuracy using SVM on MRI data, while (Richhariya et al., 2020) achieved 90% accuracy using Universum SVM (USVM) on structural MRI data.
- SVM models have also been effective in combining MRI and cognitive data, with accuracies reaching 80.9% (Arco et al., 2021). SVMs are particularly effective for small datasets and can handle both linear and non-linear relationships, but they require careful parameter tuning and can be computationally expensive for large datasets.

**K-Nearest Neighbor (KNN)**:

- KNN models are computationally simpler, but have shown good performance in AD classification. The study (KP and Thiyagarajan, 2022) used a fusion-based algorithm that combined Fisher Score and greedy search with KNN, achieving 91% accuracy on multimodal data from the ADNI and AIBL databases.
- KNN is effective for small datasets and can handle multimodal data well, but it is sensitive to feature scaling and can be computationally expensive for large datasets.

**Ensemble Models**:

- Ensemble learning techniques, which combine multiple models, have shown improved performance over single models. The study (Qu et al., 2021) used XGBoost, an ensemble method, to classify AD based on Diffusion Tensor Imaging (DTI) data, achieving 82.35% accuracy.
- Ensemble models have also been effective in handling large datasets and optimizing feature selection (Ryu et al., 2020). These models are robust to overfitting and can combine the strengths of multiple models, but they are complex to implement and computationally intensive. Table 12 represents the advantages and disadvantages of different ML models.

**Table 12:** Benefits and drawbacks of different machine learning models.

Model type	Strengths	Weakness	Reason for success and failure in AD classification
Support vector machine (Kishore et al., 2021; Arco et al., 2021)	It is effective for small datasets and good at separating linear classes	It struggles with large, high-dimensional imaging data	Works well with extracted features (e.g., hippocampus volume) but lacks depth for full MRI images
Random forest (Khan and Zubair, 2022; Parameswari et al., 2022)	It handles non-linearity well and is robust to overfitting	It requires feature selection and is expensive	Useful for tabular clinical data but struggles with complex imaging
Regression models (Sharma and Dey, 2021; Şeker et al., 2021)	It is simple, interpretable, and effective for linear relationships	Struggles with high-dimensional neuroimaging data	Useful for basic risk prediction but lacks in feature extraction for complex images
K-nearest neighbor (KP and Thiyagarajan, 2022)	Non-parametric, effective for small datasets as well as simple to implement	Computationally expensive for large datasets and also sensitive to noise	Works well for structured data but struggles with high-dimensional MRI and PET images
Decision tree (Sudharsan and Thailambal, 2021; Lin et al., 2021)	It is easy to interpret and good for tabular clinical data	Prone to overfitting and lacks robustness for complex feature interactions	It is effective for structured patient records but less accurate on raw imaging data
Ensemble models (Qu et al., 2021; Ryu et al., 2020)	Combines strengths of multiple models and it improves generalization	Complexity is higher, and it requires fine-tuning	It often achieves higher accuracy by combining DL feature extraction and traditional ML classifiers

## 6 Study on deep learning models for detecting Alzheimer's disease

Traditional ML approaches depend on handcrafted feature extraction in which relevant features are first derived from neuroimaging or clinical data and then used by classifiers such as SVM, Random Forest, or KNN for prediction. Conversely, DL models automatically learn hierarchical features directly from raw data, eliminating the need for manual feature engineering. While ML methods are effective with smaller datasets and structured features, DL approaches are particularly suitable for analyzing high-dimensional imaging data such as MRI and PET, thereby enabling improved pattern recognition and classification performance. This section presents various DL models in the detection and classification of AD.

### 6.1 Artificial neural network

An Artificial Neural Network is a network of connected nodes that receive inputs and deliver outputs based on their predefined activation function. It is used in speech recognition and image recognition. This section presents different Artificial neural networks used for the classification of Alzheimer's disease. Frank Rosenblatt implemented the first ANN in 1958 (Rosenblatt, 1958). Mathematical representation of ANN is illustrated in Equation 7.


$$\begin{array}{l} y=f\left(\sum_{i=1}^{n}\;w_{i\;}x_{i\;}+b\right) \end{array}$$ (7)

Where y denotes the output of the neuron; f denotes the activation function; w_i_ are the weights; x_i_ are the inputs; b are the bias term.

**Figure 16:**
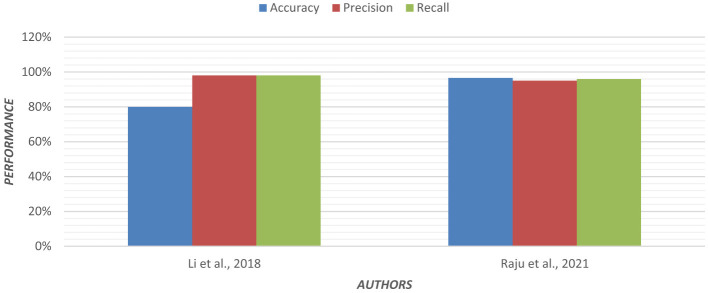
Graphical representation of the performance of ANN.

An effective connectivity network modeling method and an elastic multilayer perceptron classifier (EMLPC) were used to address the issue of overfitting in MCI detection (Li et al., 2018). In this ultra-time series, the original regional mean time series was used to produce the weak derivatives of the fMRI data. The Ultra-group constrained Orthogonal Forward Regression (UOFR) structure detection algorithm was used to determine the topology of efficient connection networks. The effective connectivity strength was computed using the UOFR technique. EMPLC was used to classify AD, and topological properties were retrieved from effective connection networks. Figure 16 shows a graphical representation of the performance of the ANN in AD classification.

For multilevel classification, sMRI images were preprocessed for Motion correction, Non-Uniform Intensity Normalization, and Skull Stripping (Raju and Gopi, 2021). The MRI picture after processing was divided into 27 patches. The complicated and basic features linked to atrophy were then extracted from these patches using various cascaded 3DCNN techniques. Once all features from various patches were combined, a multilayered perceptron was used to classify the data into AD, MCI, and NC. This model could be extended with multimodal images. Table 13 describes the contributions of Artificial Neural Networks used in the classification of AD.

**Table 13:** Review of existing research on artificial neural network.

References	Main research and contributions
Li et al. (2018)	The ultra-group constrained orthogonal forward regression (UOFR) algorithm determines the topology of effective connectivity networks, with EMLPC classifying Alzheimer's disease based on the retrieved topological properties.
(Raju and Gopi, 2021)	Structural MRI (sMRI) images are divided into patches, from which complex and simple atrophy-related features are extracted using cascaded 3D convolutional neural networks (CNNs). A multilayer perceptron (MLP) then classifies the combined features.

### 6.2 Multilayer perceptron

The Multilayer Perceptron is a fully-connected feed-forward neural network. It comprises an input, a hidden, and an output layer. It is widely used for prediction problems. In 1958, Frank Rosenblatt laid the foundation for this modern feed-forward network. Mathematical representation of MLP is illustrated in Equation 8.


$$\begin{array}{l} y=\sigma \left(\sum_{i=1}^{n}\;w_{i\;}x_{i\;}+b\right) \end{array}$$ (8)

Where y denotes the output; σ denotes the activation function; w_i_ denotes the weights; x_i_ denotes the inputs; b denotes the bias term.

**Figure 17:**
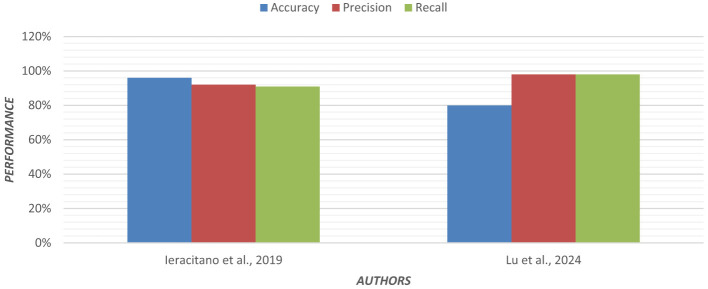
Graphical representation of performance of MLP.

In this, an electrical map of the human brain was generated by an electroencephalogram (EEG). EEG was a low-cost method with high temporal resolution. Neural circuit interactions were non-linear, non-stationary, and multivariate. The author proposed a novel multimodal ML-based approach for the automatic classification by integrating brain features (Ieracitano et al., 2020). The extracted features were obtained using BiSpectrum (BiS) and Continuous Wavelet Transform (CWT). In preprocessing, artifacts in the signal were removed, and the signal was partitioned into segments. CWT and BiS features were extracted for each segment, and vectorized features served as the multimodal input to the ML system. Four classifier models, namely Support Vector Machine (SVM), Logistic Regression (LR), Multilayer Perceptron (MLP), and AutoEncoder (AE), were trained over three combinations of features: CWT features, BiS features, and both CWT and BiS features. Among the four models, MLP yielded the best accuracy. Figure 17 depicts the graphical representation of the performance of MLP in AD classification.

In recent times, many unimodal and multimodal models have been used for classification and prediction. Though multimodal has many advantages, there is a chance of losing important information by concatenating or maximizing extracted information. To resolve this issue, Hierarchical Attention-based Multimodal Fusion (HAMF) was proposed. In this three-modality setting, data were preprocessed, and features were selected (Lu et al., 2024). Relationships among the selected features were captured by Stacked Denoising AutoEncoder (SDAE). After fusion, a two-layer MLP was used for classification. Table 14 describes the contributions of Multilayer Perceptron used in classification of Alzheimer's disease.

**Table 14:** Review of existing research on multilayer perceptron.

References	Main research and contributions
Ieracitano et al. (2020)	The proposed multimodal machine learning approach utilizes electroencephalogram (EEG) data for automatic classification of brain states by integrating features extracted from BiSpectrum (BiS) and continuous wavelet transform (CWT)
Lu et al. (2024)	This method preprocesses three modalities and selects relevant features, capturing relationships among them using a Stacked Denoising AutoEncoder (SDAE). After feature fusion, a two-layer multilayer perceptron (MLP) is employed for classification

### 6.3 Deep neural network

A Deep Neural Network is an Artificial Neural Network with layers of nodes, like the human brain, which is made of neurons. It is used to learn features from data and image classification. This section presents different Deep neural networks applied for the classification of Alzheimer's disease. The following Equation 9 is a building block for complex neural network architectures.


$$\begin{array}{l} \text{y}=\text{f}\left({\text{w}}_{\text{x}}+\text{b}\right) \end{array}$$ (9)

Where x denotes the input vector; w denotes the weight matrix; b denotes the bias vector; f denotes the activation function.

To improve the quality of the sMRI image, a gadolinium chemical substance was injected into the subject's body before taking the MRI (Soundarya et al., 2021). Brain tissue shrinkage is a major feature, and this Gadolinium substance was used to easily identify affected regions of the brain. In this approach, the Deep Neural Network achieved higher accuracy than ML models. With GPU support, a greater number of data points and hidden layers can be allowed for better accuracy.

**Figure 18:**
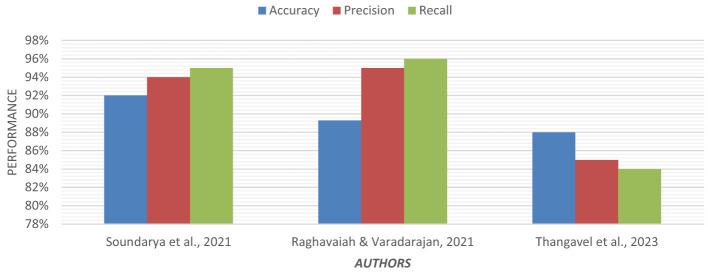
Graphical representation of the performance of DNN.

In computer-aided diagnosis, MRI data were preprocessed using the Statistical Parameter Mapping (SPM) tool. Gray matter (GM), white matter, and cerebrospinal fluid (CSF) were extracted from standardized images. The texture features were extracted using the Gabor filter, which has three scales and eight orientations (Raghavaiah and Varadarajan, 2021b). Finally, utilizing a Deep Neural Network (DNN), the retrieved features were classified. The Enhanced Squirrel Search Algorithm (ESSA) handled the classifier's weight complexities. Figure 18 shows the graphical representation of the performance of DNN in AD classification.

A deep Neural Network was used to predict AD earlier, and it uses an MRI dataset in Comma-Separated Value (CSV) format. In this EAN-DNN (Early AD-DNN) method, a deep ResNet and a deep CNN were used to train the MRI dataset (Thangavel et al., 2023). The modified Adam optimizer was used to select the most informative features from MRI data for prediction. Table 15 describes the contributions of Deep Neural Network used in classification of Alzheimer's disease.

**Table 15:** Review of existing research on deep neural network.

References	Main research and contributions
Soundarya et al. (2021)	Gadolinium injection before structural MRI (sMRI) enhances image quality and helps identify affected brain regions due to tissue shrinkage. deep neural networks, supported by GPUs, outperform traditional machine learning models in accuracy.
Raghavaiah and Varadarajan (2021a)	MRI data is preprocessed using SPM to separate GM, WM, and CSF, followed by Gabor filter-based texture feature extraction. A deep neural network classifier, optimized by the enhanced squirrel search algorithm, is used for the final classification.
Thangavel et al. (2023)	The EAN-DNN (early Alzheimer's disease-deep neural network) method utilizes deep ResNet and deep CNN architectures to predict Alzheimer's disease using MRI datasets in CSV format.

### 6.4 Convolutional neural network

A Convolutional Neural Network (CNN) is a DL architecture that directly learns from data and eliminates the need for manual feature extraction. It reduces high dimensionality of images without losing its information. It is used for image recognition and classification. The first successful convolution network was created by Professor Yann Lecunn in late 1990 LeCun and Bengio (1998). A Mathematical representation of CNN is illustrated in Equation 10.


$$\begin{array}{l} \text{Output}\left(\text{i},\text{j}\right)=\sum m\sum n\;Input\left(i+m,j+n\right)\;X\;Filter\left(m,n\right) \end{array}$$ (10)

Where output(i,j) is the value of the output feature map at position(i, j), Input(i, j) is the value of the input image at position(i, j), and Filter(m, n) is the value of the filter at position(m, n).

The author has proposed that connections among features from different layers were combined using a connection-wise attention mechanism (CAM) (Zhang et al., 2021). Each cube of the feature map was replaced with the maximum value in max pooling, and the most important features were used for classification. Skull stripping and spatial normalization were applied to the original images for better enhancement. From the preprocessed image, to extract the multiscale features, a densely connected CNN was applied. To reduce the complexity, a small-sized patch was generated from the original image. Size of the patch can be reduced to solve convergence problem.

Image restoration technique 2D Adaptive Bilateral Filter was used to remove unwanted noise from MRI images and its quality was enhanced using Adaptive Histogram Adjustment (AHA) algorithm (Sathiyamoorthi et al., 2021). Adaptive Mean Shift Modified Expectation Maximization (AMS-MEM) was used to segment the ROI. From preprocessed image 2D Gray Level Co-occurrence Matrix (GLCM) was used to find the features. In classification, the Deep Convolutional Neural Network (DCNN) had better accuracy. Adaptive clustering can improve segmentation.

Compared to single model, multi-model has good performance (Liu et al., 2020). Multi task deep CNN was used for the segmentation of hippocampus. From the segmented image 3D patches were extracted and features were learned using 3D DenseNet. The selected features from deep CNN and 3D DenseNet were trained individually and then combined using fully connected layer for classification. Similarly, 3D shearlet based descriptors and fine-tuned pretrained CNN gets the average and compact features for each MRI specimen (Alinsaif et al., 2021). CNN and 3D shearlet descriptor features were integrated and then trained using two classifiers namely SVM, DTB (Decision Tree Bagger). Classification based on combining models had better accuracy than the other. Techniques used in classification can be used in segmentation.

Significant characteristics were retrieved from axial, sagittal and frontal positions of MRI images using AlexNet framework (Kumar et al., 2022). Transfer learning with formulated AlexNet was to find out the best abstractions of data for classification. This existing well-tuned model improves the performance. The model can be tuned to use amyloid protein aggregation as a predictor for AD. Transfer learning with GoogleNet achieved higher accuracy than Deep Separable Convolution (DSC). This DSC was replacement of conventional convolution Net (Liu et al., 2021). The generalization performance of this model can be improved. Sagittal MRI images were fed to the ResNet ANN to extract features. Subject's age and sex were concatenated with the features (Puente-Castro et al., 2020). The data were trained using SVM classifier and Transfer learning was used for its size and better classification. In future sagittal, information can be combined with other plane information to improve the prediction accuracy.

In AD diagnosis, the Genetic Programming (GP) model involved a majority voting-based scheme to select the significant features (Nasrolahzadeh et al., 2022) used for classification. From the input speech signal, features were extracted based on higher-order spectra. And based on the features, models were developed using symbolic regression and feature construction. AD may cause loss in brain regions like Gray Matter (GM), White Matter (WM), Corpus Callosum (CC), and Hippocampus (HC). AD can be diagnosed from the sub-regions of the brain using optimization algorithms like Genetic Algorithm (GA), Gray Wolf Optimization (GWO), and Cuckoo Search (CS; Chitradevi and Prabha, 2020). Images were segmented and validated with ground truth images, and the GWO optimization method had better accuracy.

**Figure 19:**
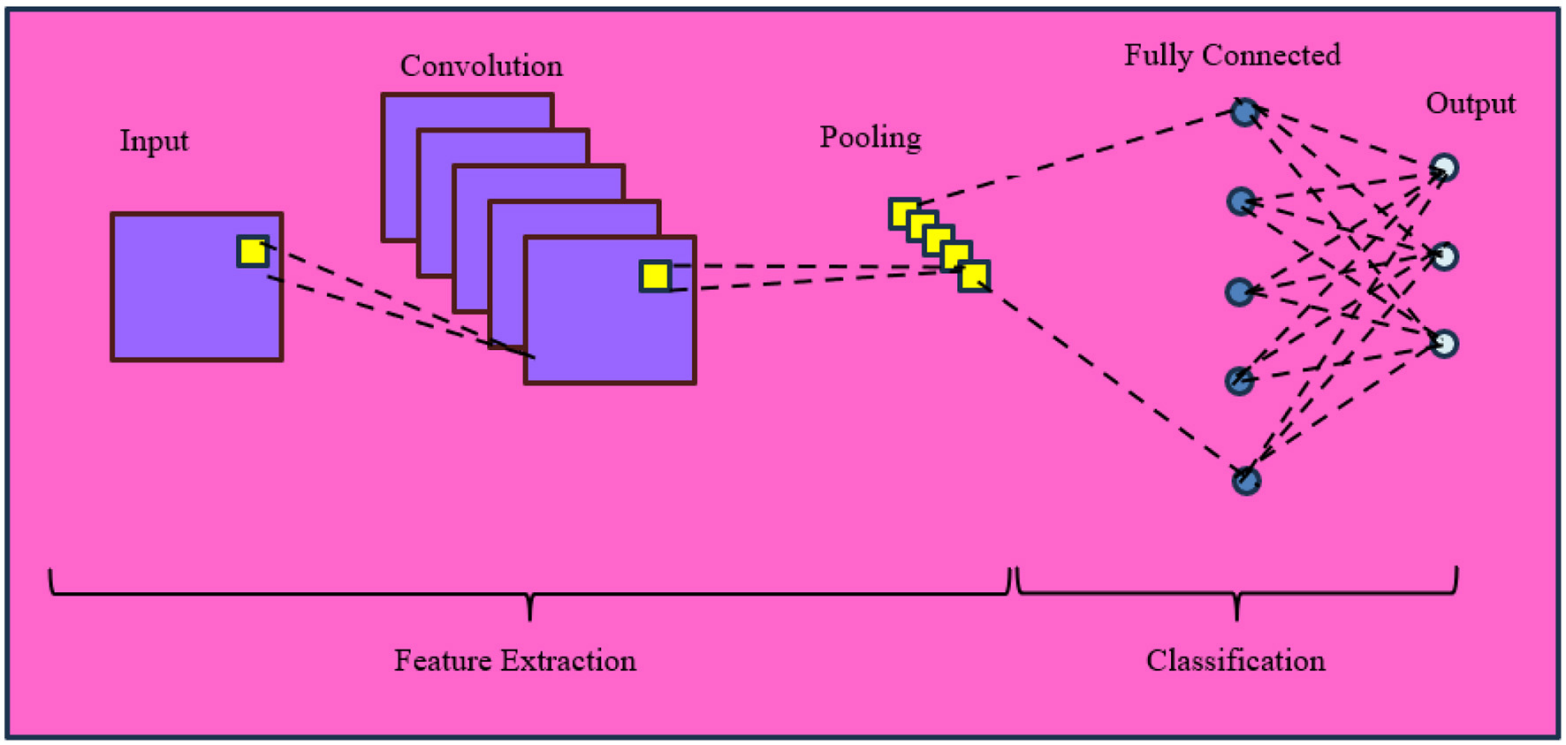
Basic architecture of CNN.

To discriminate AD, one slice of input data has only limited information. So, the features were extracted from three slices namely axial, coronal, sagittal view and center of hippocampus were fed to the PCANet (Bi et al., 2020). These three feature vectors were concatenated to obtain the final features, which were then used as inputs to K-means clustering to improve prediction results. A deep CNN VGG-16 was used for feature extraction, and high-level features extracted were given to classifiers (Janghel and Rathore, 2021). To improve the accuracy, input images were converted from 3D to 2D, segmented, and then fed into the model. SVM, Linear discriminant, K-means clustering, and Decision tree were the four classifiers used. Among them, SVM achieved better accuracy. It can be extended to reduce execution time. Figure 19 illustrates the different layers of CNN and their role in feature extraction and classification.

Features are essential for categorization but different dimensions of data leads to complication in diagnosis. So, input 3D images were preprocessed and ensemble of 11 Auto encoders were grouped to extract features and the features can be enhanced by adding one layer in encoder space (Hedayati et al., 2021). Then it was fed to the CNN classifier. Optimized and adaptive filters in this structure help handle different types of data. Another fully automated DL approach had three trained networks, which were grouped together namely Alex Net, ResNet-101 and Inception ResNet-V2 (Loddo et al., 2022). After fine tuning the networks, features were extracted and average was calculated. Based on the average ensemble bagged tree classifier performed the classification. Compared to single model, grouping of three models had better accuracy.

3D images were converted to 2D image slices for training a 2D CNN, and it was difficult to find temporal dependencies. To address this issue and improve the parallel processing, a sequence-based model called Temporal Convolutional Network (TCN) was used (Ebrahimi et al., 2021). It performed both feature extraction and classification simultaneously and had higher accuracy. Compared to 3D CNN, it requires fewer resources. The structure of the TCN can be improved.

For easy processing, 4D input images were sliced into 2D/3D images, resulting in a loss of information. To avoid this loss of 4D images, the DL model C3d-LSTM was proposed with two modules, namely 3D CNN and LSTM (Li et al., 2020). The first module 3D CNN is for extracting spatial features from input images and extracted features were fed into Long-Short-Term-Memory for prediction.

The inputs obtained from Magnetoencephalography have a larger number of features than the number of samples. To avoid this overfitting limitation same sub models were grouped and in final stage average was calculated across the sub models (Lopez-Martin et al., 2020). Each model had interpreted 2D images with randomly ordered features. This randomized 2D CNN performed better. DL models and stochastic methods can be combined for better classification.

3D sMRI sagittal plane gives more visual features of the mid-brain regions. Preprocessing includes normalized to specific standards, realignment of images and image registration (Sharma et al., 2022). Features were extracted from sagittal plane using deep residual network (ResNet-101). Extracted features were given to Fuzzy hyper plane based Least Square twin support vector machine (FLS-TWSVM) and it attained better accuracy.

**Figure 20:**
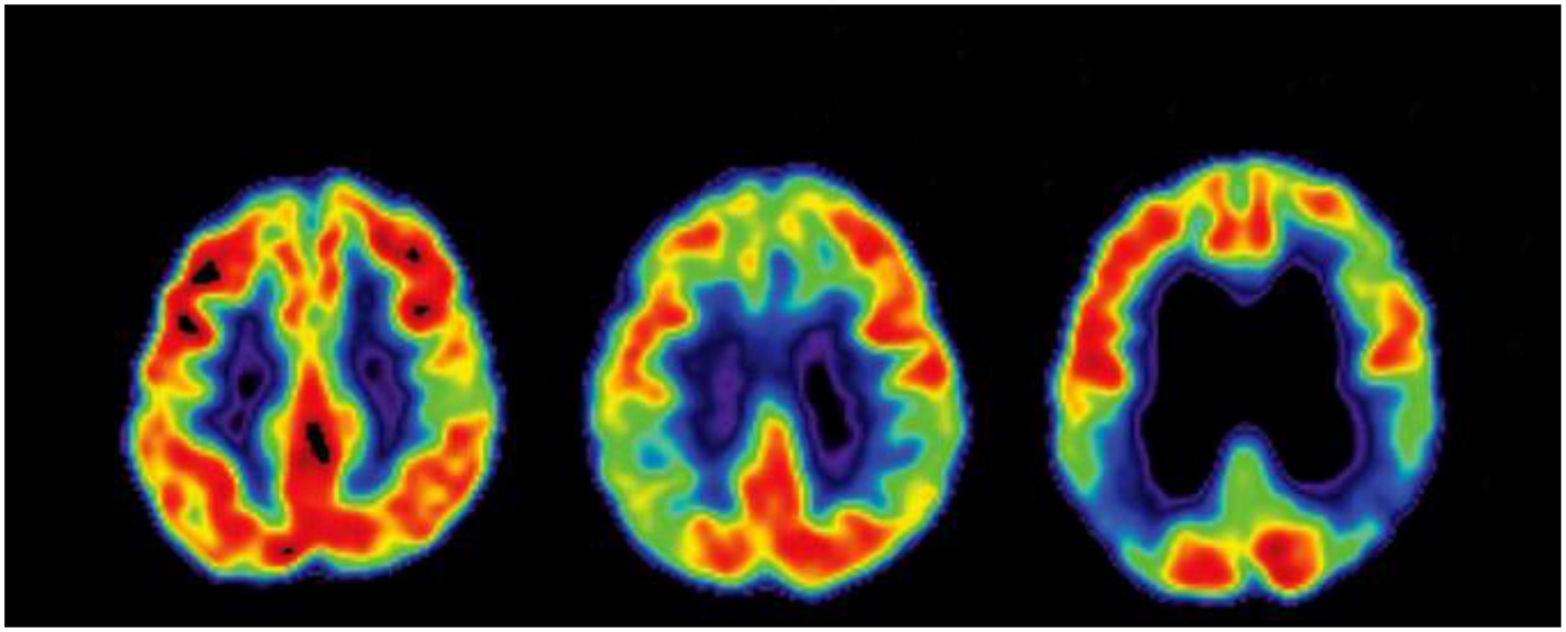
Sample PET images of the brain.

Feed forward Local Phase Quantization Network (LPQNet) model has phases like feature generation, feature selection and classification (Kaplan et al., 2021). In feature generation phase the loaded MRI images were converted to gray scale images. LPQ was applied to obtain first 256 features and average pooling was deployed to generate decomposed images. From the decomposed images, features were extracted. The extracted feature vectors were merged to get the final feature vector. In the selection phase, 256 most discriminative features were selected using Neighborhood Component Analysis (NCA). In the classification phase, Decision Tree, Linear Discriminant, Logistic Regression, Naive Bayes, SVM, KNN, and Bagged Tree were the seven classifiers trained to obtain the best accuracy. Figure 20 shows the PET images of Cognitively Normal, Mild Cognitive Impairment, and AD subjects.

In DL, three slices, namely axial, sagittal, and coronal MRI images, were used in multiclass classification. Preprocessing involves oversampling, undersampling, processing, normalization, resizing, denoising, and format conversion. Preprocessed images were augmented using rotation and reflection techniques (Helaly et al., 2022). For classification, variants of CNNs and pretrained VGG-19 model were trained to get the better accuracy and it was evaluated using nine performance metrics.

The iterative Sparse and Deep Learning (ISDL) model was a combination of Deep Feature Extraction (DFE) and a sparse regression module to extract the features and diagnose AD (Chen and Xia, 2021). In the DFE module, ResNet10 was used as sub model, followed by a collaborative layer that permitted collaborative training of all sub models. The sparse regression module based on the obtained feature matrix performs the diagnosis of MCI and AD. Sparse features and sparsity in data can be explored.

The process of transferring knowledge from a large model to a smaller one is termed knowledge distillation. Due to the cost complexity of multimodal data, MRI-based prediction multi-modal multi-instance distillation was proposed by deploying knowledge distillation (Guan et al., 2021). The large model is a teacher network that contains multimodal data. The smaller model is a student network contained only one data. Within the teacher-student network, knowledge distillation takes place, and after training, a student network can accurately categorize AD using just MRI data.

**Figure 21:**
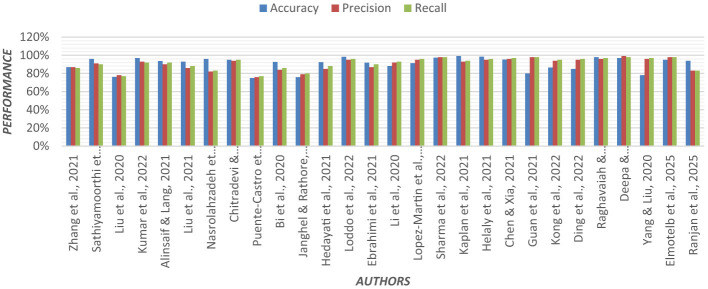
Graphical representation of performance of the CNN.

For better classification, MRI and PET images were fused to get richer feature information (Kong et al., 2022). Both structural MRI and functional PET images were preprocessed before the fusion. In MRI image preprocessing, like skull stripping, standardization and registered to specific template. PET images also standardized and registered to specific template. After registration MRI and PET images will have same properties and mapping was applied to obtain a fused image. Auto encoder was used to extract features from the images and then 3D CNN was trained for classification. Figure 21 shows the graphical representation of the accuracy achieved by CNN in classification.

The conventional method for AD detection is a ROIs-based multi-resolution 3D-HOG feature learning approach. MRI images were preprocessed into ROI-based images for future processing (Ding et al., 2022). The first module was a multi-resolution 3D-HOG feature extraction method that accurately depicts the properties of the image by describing both regional and global texture changes for AD detection. The second module was the histogram-based wrapped feature selection method that can identify unique subareas of ROIs for AD detection in addition to selecting discriminative histograms with high efficiency.

To detect AD from magnetic resonance imaging (MRI) and functional MRI (fMRI), state-of-the-art DL-formed pipelines were used (Raghavaiah and Varadarajan, 2021a). The data was meticulously preprocessed using these pipelines, which were executed on a GPU-based processing stage. The preprocessed image was then transformed into a PNG image in the image transformation block. Then, scale-and motion-invariant low-to-unmistakable-intensity highlights were obtained for convolutional neural network usage. For classification, a CNN classifier was utilized.

The DL platform utilized Convolutional Architecture for Fast Feature Embedding (CAFFE) as a framework for transformation prediction (Yang and Liu, 2020). Images that have already been analyzed were converted to Lightning Memory-Mapped Database (LMDB) format. The pre-trained CAFFE model was used in the feature extraction process. After feature extraction, the feature rows were displayed using the network model's output parameters for every tier. For the EMCI and LMCI classification models, a feature classification set was created, and the predictive model for MCIc and MCInc was created. The SFS algorithm was used to choose the training set after the PCA was used to reduce the dimension. Finally, a classifier is used for classification.

The optimization of VGG-16 architecture with Arithmetic Optimization Algorithm (AOA) for AD classification was proposed to address the computational cost and lack of multimodal data corporation (Deepa and Chokkalingam, 2022). Pre-processing, segmentation, and classification were the three main modules. During pre-processing, the format of T1-weighted MRI images was processed using the CAT12 toolbox. While the linear contrast stretching raises the image contrast level, image-enhancing techniques normalize the unequal light distribution. Lastly, an Optimized VGG-16 with AOA successfully differentiates between normal, moderate dementia (severe cognitive decline), and late dementia (extremely severe cognitive decline) classes of AD. Table 14 presents the contributions of Convolutional Neural Network used in classification of Alzheimer's disease.

A DL approach using ResNet152V2 with a novel hyperparameter optimization (HPO) model for multi-class AD classification (Mild Dementia, Moderate Dementia, Normal, Very Mild Dementia; Elmotelb et al., 2025). It was tested on a Kaggle MRI dataset, and the model outperformed transfer learning and classical models in precision, recall, F1-score, and accuracy, addressing data and computational limitations for early AD detection. A DL-based approach for dementia classification (AD, MCI, HC) using EEG-derived scout time-series signals from subcortical regions (hippocampus, amygdala, thalamus; (Ranjan et al., 2025). Signals were converted to images via continuous wavelet transform (CWT) and classified using DenseNet, achieving 94.17% accuracy (BrainLat dataset: 16 AD, 13 FTD, 19 HC) and 77.72% (IITD-AIIA dataset: 10 AD, 9 MCI, 8 HC). The method leverages sLORETA and probability-based classifier fusion, highlighting potential for early, accurate dementia diagnosis. Table 16 illustrates the contributions of CNN in AD classification.

**Table 16:** Review of existing research on convolutional neural networks.

References	Main research and contributions
Zhang et al. (2021)	The proposed method utilizes a connection-wise attention mechanism (CAM) to enhance feature connections across different layers for Alzheimer's disease classification.
Sathiyamoorthi et al. (2021)	The 2D adaptive bilateral filter is used to remove noise from MRI images, enhanced by the adaptive histogram adjustment (AHA) algorithm, while adaptive mean shift modified expectation maximization (AMS-MEM) segments the region of interest (ROI). Features are extracted using the 2D Gray level co-occurrence matrix (GLCM), and classification is performed with a deep convolutional neural network (DCNN).
Liu et al. (2020)	The proposed method utilizes a multi-task deep CNN for segmenting the hippocampus from MRI images. Multi-model approaches demonstrate superior performance in Alzheimer's disease diagnosis.
Kumar et al. (2022)	The proposed method utilizes the AlexNet framework to extract significant characteristics from axial, sagittal, and frontal positions of MRI images for Alzheimer's disease classification.
Nasrolahzadeh et al. (2022)	The genetic programming (GP) model for Alzheimer's disease diagnosis uses a majority voting-based scheme to select significant features extracted from input speech signals based on higher-order spectra.
Chitradevi and Prabha (2020)	Alzheimer's disease (AD) can be diagnosed by analyzing the sub-regions of the brain using optimization algorithms like genetic algorithm (GA), gray wolf optimization (GWO), and Cuckoo search (CS).
Puente-Castro et al. (2020)	Sagittal MRI images are utilized in a ResNet artificial neural network (ANN) to extract relevant features for Alzheimer's disease diagnosis.
Bi et al. (2020)	To discriminate Alzheimer's disease (AD), features are extracted from axial, coronal, and sagittal MRI slices, focusing on the center of the hippocampus, and fed into a PCANet. The concatenated feature vectors are then input into K-means clustering to enhance prediction accuracy.
Sharma et al. (2022)	The proposed method focuses on utilizing 3D structural MRI (sMRI) images in the sagittal plane to extract visual features from mid-brain regions for Alzheimer's disease diagnosis.
Kong et al. (2022)	MRI and PET images are fused to obtain richer feature information for Alzheimer's disease classification, with preprocessing steps including skull stripping, standardization, and registration to a specific template.
Ding et al. (2022)	The conventional method for Alzheimer's disease (AD) detection utilizes a regions of interest (ROI)-based multi-resolution 3D-HOG feature learning approach.
Deepa and Chokkalingam (2022)	The proposed method optimizes the VGG-16 architecture using the arithmetic optimization algorithm (AOA) for Alzheimer's disease (AD) classification, addressing computational cost and lack of multimodal data integration.
Elmotelb et al. (2025)	This deep learning approach using ResNet152V2 with a novel hyperparameter optimization (HPO) model was used for multi-class Alzheimer's disease (AD) classification.
Ranjan et al. (2025)	This is a deep learning-based approach for dementia classification (AD, MCI, HC) using EEG-derived scout time-series signals from subcortical regions.

### 6.5 Recurrent neural network

A recurrent neural network is a type of artificial neural network, mainly used for sequence classification. This section presents different RNNs used for the classification of Alzheimer's disease. In 1924 the first RNN architecture was developed by Ising (1924). Mathematical representation of RNN is illustrated in Equation 11.


$$\begin{array}{l} \text{h\_t}=\text{f}\left({\text{W}}^{*}\text{x\_t}+{\text{U}}^{*}\text{h\_}\left\{\text{t}-1\right\}+\text{b}\right) \end{array}$$ (11)

Where h_t is hidden state at time step t, f is activation function, U and W are weight metrics, b is the bias vector, x_t is input at time step t.

From the preprocessed data, features required for classification were selected using two trees based and regularization based embedded feature selection models. Based on the importance of feature AdaBoost was selected as best model for feature selection. Proposed model Enhanced Deep Recurrent Neural Network (EDRNN) process the selected features (Mahendran and PM, 2022) and gives better accuracy in classification.

**Figure 22:**
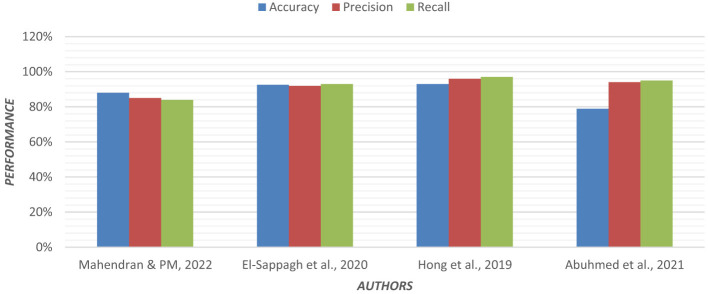
Graphical representation of performance of RNN.

Multimodal time series data like MRI, PET, Cognitive scores, Neuropathology data, Assessment data and Background knowledge was fused (El-Sappagh et al., 2020). For MRI and PET, Principal Component Analysis was used to extract principal components. In Parallel background knowledge data also preprocessed. CNN-BiLSTM model was used to train time series data. Then both the features were concatenated to extract common features and then second decision fusion was made to get abstract common features. In final step task specific features were learned by dense layers and sigmoid or SoftMax was used for classification. Figure 22 presents the graphical representation of performance of RNN in AD classification.

A recurrent neural network called Long Short-Term Memory (LSTM) was applied to forecast the growth of AD (Hong et al., 2019). Skull stripping, registration, segmentation, normalization, and smoothing were the major steps in pre-processing of MRI images. Pre-Fully Connected Layer, Cells Layer, and Post-Fully Connected Layer were present in the suggested model. One fully connected layer and a ReLU function make up the Pre-Fully Connected layer, One LSTM layer and a Dropout Wrapper make up the Cells layer, one fully connected layer and a SoftMax layer make up the Post-Fully Connected Layer. Time series data and brain area features were fed to the model, and the pre-fully connected layer extracts the most relevant combinations of information. The Cells layer chooses the time-sensitive characteristics, which are then sent to the Post-Fully Connected layer, which uses the combinations of the time-sensitive features to predict AD.

Hybrid DL models were used to tackle the limitations in handling multimodal data (Abuhmed et al., 2021). In the first HDL model, DFBL (Deep Feature Based Learning) included a multivariate BiLSTM architecture for learning deep feature representations, and a standard ML model for classification, such as a Decision Tree, Random Forest, or Support Vector Machine. The second HDL model, called MRBL (Multi-task Regression Based Learning), has two main phases. Seven regression tasks are jointly learned using a multivariate BiLSTM model in the first phase, and the multiclass AD progression was learned using a conventional ML model in the second phase. DL and ML methods, namely the SoftMax classifier, RF, SVM, DT, general model (GM), FURIA, and Multi-Objective Evolutionary Fuzzy Classifier (MOEFC), were employed based on a set of cognitive scores. Table 17 presents the contributions of Recurrent Neural Network used in classification of Alzheimer's disease.

**Table 17:** Review of existing research on recurrent neural network.

References	Main research and contributions
Mahendran and PM (2022)	Feature selection using tree-based and regularization-based embedded models, with AdaBoost as the best approach, precedes the processing of selected features by an enhanced deep recurrent neural network (EDRNN) for improved classification accuracy
El-Sappagh et al. (2020)	In this study, principal component analysis is used to extract principal components, and a CNN-BiLSTM model is used to train time series data. Task-specific features are learned by dense layers, and sigmoid or SoftMax is used for classification
Hong et al. (2019)	The model comprises a pre-fully connected layer, a cells layer with LSTM, and a post-fully connected layer, which together extract relevant features and predict AD based on time-sensitive characteristics
Abuhmed et al. (2021)	DFBL (deep feature based learning) included a multivariate BiLSTM architecture for learning deep feature representations, and a standard machine learning model for classification

### 6.6 Generative adversarial networks

Generative Adversarial Networks (GAN) is a neural network used for unsupervised learning. It is made up of two neural networks namely, discriminator and generator. This concept was developed by Goodfellow et al. (2014). Mathematical representation of GAN is illustrated in Equation 12.


$$\begin{array}{l} \text{V}(\text{D},\text{G})=\text{Ex}\sim \text{pdata}(\text{x})[\text{logD}(\text{x})]+\text{Ez}\sim \text{pz}(\text{z})[\text{log}(1\\ \;-\text{D}(\text{G}(\text{z})))] \end{array}$$ (12)

Where G is generator network, D is discriminator network, D(G(z)) is discriminators output for generated data sample.

Marine Predators Algorithm (MPA) was used for enhancing classification (Sekhar et al., 2023). GAN generates synthetic EEG samples from raw EEG data, to correct data imbalances and simulate EEG features. Features extracted by GAN were optimized by MPA for better classification. But GAN-MPA approach also has challenges like high computational demands, complex model structures.

**Figure 23:**
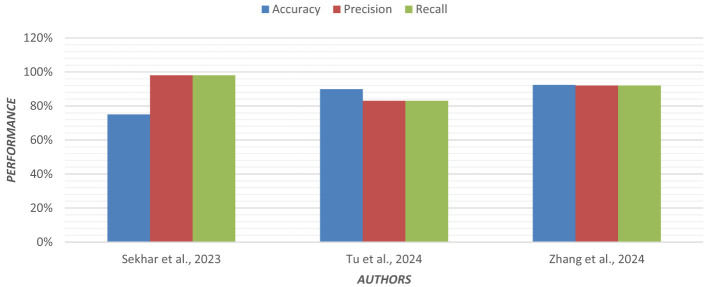
Graphical representation of the performance of GAN.

Most of the existing models exclude PET images, for its expensive, tough to use and high radiation property. To solve the challenges in multimodal fusion, Consistent Manifold Projection Generative Adversarial Network (CMPGAN) for Fluorodeoxyglucose Positron Emission Tomography (FDG-PET) generation and Multilevel Multimodal Fusion Diagnosis Network (MMFDN) for AD diagnosis was used (Tu et al., 2024). CMPGAN module consists of a generator and a discriminator. Generated samples and actual data are distinguished by discriminator. Both the generator and discriminator were trained adversarilly for optimizing the performance of the model. Complete input was fed into MMFDN module for feature level and voxel level fusion to get accurate result. Figure 23 shows the graphical representation of performance of GAN in AD classification.

Pyramid Attentive GAN generates PET images through a pyramid attention mechanism (Zhang et al., 2024) and standard discriminators to address missing PET images. PET and MRI images were fused at the pixel level. The AD diagnosis network also uses a pyramid generator to predict the multi-resolution features extracted from fused images. Table 18 shows the contributions of Generative Adversarial Network used for the classification of Alzheimer's disease.

**Table 18:** Review of existing research on GAN.

References	Main research and contributions
Sekhar et al. (2023)	The Marine Predators algorithm (MPA) has been used in combination with generative adversarial networks (GANs) to enhance EEG-based classification, particularly in addressing data imbalances and simulating EEG features
Zhang et al. (2024)	The consistent manifold projection generative adversarial network (CMPGAN) generates fluorodeoxyglucose positron emission tomography (FDG-PET) images, with a generator and discriminator trained adversarially
Tu et al. (2024)	The pyramid attentive GAN generates PET images through a pyramid attention mechanism and discriminators, enabling pixel-level fusion with MRI images

**Figure 24:**
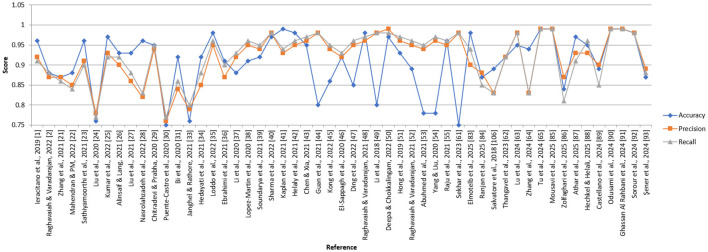
Graphical representation of performance of deep learning models.

Table 19 presents the performance of different DL models based on Accuracy, Precision, and Recall used in the literature. Figure 24 shows the graphical representation of the performance of DL models used in AD classification.

**Table 19:** Performance of different DL models.

References	Medical image	Classification model	Datasets	Comparison performed	Accuracy (%)	Precision	Recall
Ieracitano et al. (2020)	EEG	Multi-layer perceptron (MLP)	189 subjects from IRCCS	AD vs. CN	96	0.92	0.91
Raghavaiah and Varadarajan (2022)	MRI	CAD and hybrid DL	ADNI	Multiclass (CN vs. MCI vs. AD)	88	0.87	0.88
Zhang et al. (2021)	MRI	Connection wise attention mechanism-CNN	968 subjects from ADNI	Multiclass (CN vs. MCI vs. AD)	87	0.87	0.86
Mahendran and PM (2022)	MRI	Enhanced deep recurrent neural network	68 subjects	AD vs. CN	88	0.85	0.84
Sathiyamoorthi et al. (2021)	MRI	Deep convolutional neural network	Not mentioned	AD vs. CN	96	0.91	0.90
Liu et al. (2020)	MRI	Deep CNN and 3D DenseNet	439 subjects from ADNI	Multiclass (CN vs. MCI vs. AD)	76.2	0.78	0.77
Kumar et al. (2022)	MRI	AlexNet	5,000 images from OASIS	AD vs. CN	97	0.93	0.92
Alinsaif et al. (2021)	MRI	3D-Shearlet descriptors and CNN with SVM	200 subjects from ADNI	AD vs. CN	93.5	0.90	0.92
Liu et al. (2021)	sMRI	GoogleNet	416 subjects from OASIS	AD vs. CN	93	0.86	0.88
Nasrolahzadeh et al. (2022)	Speech signals	Genetic programming with Bi-FFT features	60 subjects	AD vs. CN	96	0.82	0.83
Chitradevi and Prabha (2020)	MRI	Gray wolf optimization for hippocampus region, AlexNet	200 subjects	AD vs. CN	95	0.94	0.95
Puente-Castro et al. (2020)	MRI	ResNet with SVM	1,743 from ADNI, 436 from OASIS	MCI vs. AD	75	0.76	0.77
Bi et al. (2020)	MRI	PCANet with K-means clustering	1,075 subjects	AD vs. CN	92.6	0.84	0.86
Janghel and Rathore (2021)	fMRI, PET	VGG16 with SVM	2,729 images	AD vs. CN	76	0.79	0.80
Hedayati et al. (2021)	MRI	CNN	800 subjects from ADNI	Multiclass (CN vs. MCI vs. AD)	92.5	0.85	0.88
Loddo et al. (2022)	MRI	Deep ensemble model (AlexNet, ResNet)	416 from OASIS	Multiclass (CN vs. MCI vs. AD)	98.2	0.95	0.96
Ebrahimi et al. (2021)	MRI	Temporal convolutional network	450 subjects from ADNI	Multiclass (CN vs. MCI vs. AD)	91.78	0.87	0.90
Li et al. (2020)	fMRI	3D CNN	389 subjects from ADNI	AD vs. CN	88.12	0.92	0.93
Lopez-Martin et al. (2020)	MEG	2D CNN	132 subjects	AD vs. CN	91.25	0.95	0.96
Soundarya et al. (2021)	sMRI	DNN	416 subjects from OASIS	AD vs. CN	92	0.94	0.95
Sharma et al. (2022)	sMRI	ResNet with FLS-TWSVM	400 subjects from ADNI	Multiclass (CN vs. MCI vs. AD)	97.29	0.98	0.98
Kaplan et al. (2021)	MRI	LPQNet	263 subjects	Multiclass (CN vs. MCI vs. AD)	99	0.93	0.94
Helaly et al. (2022)	MRI	CNN	300 subjects from ADNI	Multiclass (CN vs. MCI vs. AD)	98.47	0.95	0.96
Chen and Xia (2021)	MRI	Iterative sparse and deep learning	1,248 subjects from ADNI	Multiclass (CN vs. MCI vs. AD)	95.32	0.96	0.97
Guan et al. (2021)	MRI	Multi-modal multi-instance distillation	669 subjects from ADNI	AD vs. CN	80	0.98	0.98
Kong et al. (2022)	MRI, PET	3D CNN	740 subjects from ADNI	AD vs. CN	86.52	0.94	0.95
El-Sappagh et al. (2020)	MRI, PET, cognitive scores, Neuropathology data, Assessment data	CNN and BiLSTM	1,536 subjects from ADNI	AD vs. CN	92.62	0.92	0.93
Ding et al. (2022)	MRI	CNN	434 subjects from ADNI	MCI vs. AD	85	0.95	0.96
Raghavaiah and Varadarajan (2021b)	MRI, fMRI	CNN	Not mentioned	MCI vs. AD	98	0.96	0.97
Li et al. (2018)	fMRI	EMLP	73 subjects from ADNI	Multiclass (CN vs. MCI vs. AD)	80	0.98	0.98
Deepa and Chokkalingam (2022)	MRI	VGG with AOA	819 subjects from ADNI	Multiclass (CN vs. MCI vs. AD)	97	0.99	0.98
Hong et al. (2019)	MRI	LSTM	1,105 subjects from ADNI	MCI vs. AD	93	0.96	0.97
Raghavaiah and Varadarajan (2021a)	MRI	Optimal DNN	280 subjects from ADNI	AD vs. CN	89.29	0.95	0.96
Abuhmed et al. (2021)	MRI, PET, cognitive scores, Neuropathology data, Neuropsychological data	Deep BiLSTM	1,371 subjects from ADNI	AD vs. CN	78.89	0.94	0.95
Yang and Liu (2020)	PET	CNN	819 subjects from ADNI	AD vs. CN	78	0.96	0.97
Raju and Gopi (2021)	sMRI	MLP	465 subjects from ADNI	Multiclass (CN vs. MCI vs. AD)	96.6	0.95	0.96
Sekhar et al. (2023)	EEG	GAN	283 subjects	AD vs. CN	75	0.98	0.98
Thangavel et al. (2023)	MRI	EAD-DNN (deep ResNet and deep CNN)	ADNI	AD vs. CN	98	0.90	0.94
Lu et al. (2024)	MRI, PET, cognitive	Hierarchical attention MLP	ADNI	Multiclass (CN vs. MCI vs. AD)	87	0.88	0.85
Zhang et al. (2024)	MRI, PET	Pyramid-attentive GAN	370 subjects from ADNI	Multiclass (CN vs. MCI vs. AD)	89.9	0.83	0.83
Tu et al. (2024)	MRI, FDG-PET	CMPGAN + MMFDN	ADNI	AD vs. CN	92.3	0.92	0.92
Elmotelb et al. (2025)	MRI	ResNet152V2	6,400 images from Kaggle	AD vs. CN	95	0.98	0.98
Ranjan et al. (2025)	EEG	DenseNet	48 subjects from BrainLat	AD vs. CN	94	0.83	0.83
Mousavi et al. (2025)	MRI	InceptionResNetV2	6,735 MRI images	AD vs. CN	99	0.99	0.99
Zolfaghari et al. (2025)	MRI	Dual parallel CNN ensemble	ADNI	Multiclass (CN vs. MCI vs. AD)	99.06	0.99	0.99
Athar et al. (2025)	PET	Deep learning with LoG and Prewitt augmentation	ADNI	AD vs. CN	84	0.87	0.81
Hechkel and Helali, (2025)	MRI, DTI	YOLOv11	ADNI	Multiclass (CN vs. MCI vs. AD)	97	0.93	0.91
Castellano et al. (2024)	MRI, Amyloid PET	CNN	ADNI	AD vs. CN	95	0.93	0.96
Odusami et al. (2024)	MRI, PET	Quantum dynamic optimization and CNN	ADNI	Multiclass (CN vs. MCI vs. AD)	89	0.90	0.85
Ghassan Al Rahbani et al. (2024)	MRI	ResNet	Public MRI datasets	Multiclass (CN vs. MCI vs. AD)	99.41	0.99	0.99
Sorour et al. (2024)	MRI	CNN-LSTM	Kaggle MRI dataset	Multiclass (CN vs. MCI vs. AD)	99.92	0.99	0.99
Sener et al. (2024)	MRI	EfficientNetB0 (McNemar's test)	ADNI	Multiclass (CN vs. MCI vs. AD)	98	0.98	0.98
Salvatore et al. (2018)	MRI	SVM with pattern recognition	540 subjects from ADNI	MCI-to-AD conversion	87	0.89	0.88

### 6.7 Comparative analysis of deep learning models

This section provides a detailed summary of the reviewed studies and offers a comparative analysis of the performance of different DL models in AD diagnosis. The goal is to highlight the strengths, limitations, and potential of these models in improving the accuracy and reliability of AD diagnosis. Table 20 presents the strength and weakness of the models and also the reason for their success and failure.

**Table 20:** Advantages and disadvantages of DL models.

Model type	Strength	Weakness	Reason for success	Reason for failure
Convolutional neural network (CNN; Zhang et al., 2021; Sathiyamoorthi et al., 2021; Liu et al., 2020; Kumar et al., 2022; Liu et al., 2021; Janghel and Rathore, 2021; Loddo et al., 2022; Helaly et al., 2022; Kong et al., 2022) etc.	It has an automatic learning ability of hierarchical features from raw images, excellent spatial pattern recognition, and transfer learning enables high performance on small datasets.	High computation, prone to overfitting without augmentation.	It captures volumetric and textural changes missed by hand-crafted features.	Depends on large labeled data; sensitive to label noise in longitudinal ADNI and poor generalization without domain adaptation.
Recurrent neural network/LSTM (Mahendran and PM, 2022; El-Sappagh et al., 2020; Hong et al., 2019)	Good for time-series MRI or cognitive data.	It requires a large memory and is less effective on purely spatial data.	Captures disease progression patterns.	Struggles with very high-dimensional 3D/4D volumes, mostly combined with CNN.
Generative adversarial networks (GAN; Sekhar et al., 2023; Zhang et al., 2024; Tu et al., 2024)	It generates synthetic MRI/PET to solve data scarcity and imbalance, and enables cross-modal synthesis.	Instability in training, high computational cost, and generated images may introduce artifacts.	It reduces PET costs and radiation exposure.	Variation in the quality of synthetic data can degrade accuracy if not carefully validated.
Transformer-based models (mentioned in ensembles Chen et al., 2023)	Global attention captures long-range dependencies and is superior to CNNs on sequence or patch-based imaging data.	It requires extremely high computation, and it requires large amounts of data for training.	Emerging SOTA in 2024–2025, which is better at multimodal fusion.	Rarely used due to data and computation limits.
Ensemble/hybrid DL (Loddo et al., 2022; Ma et al., 2023; Chen et al., 2023; Jenber Belay et al., 2024; Kavitha et al., 2022)	It combines complementary strengths, reduces variance, and often achieves the highest accuracy.	More complexity in inference time and interpretability.	Frequently reaches 98–99%, and it is robust to noisy and imbalanced data.	Risk of overfitting to training distribution if not regularized; harder to deploy clinically.

## 7 Evaluation metrics

The evaluation of ML and DL models in AD classification relies heavily on the choice of metrics. While accuracy is the most commonly reported metric, it is often insufficient for a comprehensive evaluation, especially in imbalanced datasets or when the cost of false positives and false negatives varies. This section provides an in-depth analysis of the evaluation metrics used in the literature, including Cohen's Kappa, Precision, Recall, F1-Score, accuracy, and Root Mean Square Error (RMSE).

### 7.1 Accuracy

Accuracy is a fundamental metric that calculates the percentage of all events in the dataset that were accurately predicted (true positives and true negatives). The accuracy can be calculated using Equation 13.


$$\begin{array}{l} Accuracy=\frac{TP+TN}{TP+FN+FP+TN} \end{array}$$ (13)

It was commonly used in balanced datasets where the cost of false positives and false negatives is similar. In the regression model (Sharma and Dey, 2021) for gene expression data, 92% accuracy was achieved.

### 7.2 Precision

Precision is a critical metric that quantifies the proportion of true positive predictions among all instances predicted as positives. Precision can be calculated using Equation 14.


$$\begin{array}{l} Precision=\frac{TP}{TP+FP} \end{array}$$ (14)

It is important in the case of high false positives. The Universum SVM model (USVM) had achieved high precision (Richhariya et al., 2020) for structural MRI data.

### 7.3 Recall

Recall is also known as specificity; it is the percentage of actual positive cases among all true positive predictions. Recall can be calculated using Equation 15.


$$\begin{array}{l} Recall=\;\frac{TP}{TP+FN} \end{array}$$ (15)

In literature (Feng et al., 2020), recall was used to evaluate the performance of their wavelet transformation energy feature (WTEF) model for structural MRI.

### 7.4 F1-score

The F1-Score is another popular metric that provides an evaluation of the model's performance by combining precision and recall into a single result. F1-Score can be calculated using Equation 16.


$$\begin{array}{l} F1-Score=2\;*\;\frac{\left(precision\;*\;recall\right)}{\left(precision+recall\right)} \end{array}$$ (16)

It is useful in imbalanced datasets where both precision and recall are important. Study (Qu et al., 2021) reported the F1-score in their ensemble models for AD classification using DTI data.

### 7.5 Root-mean square error

Root Mean square Error calculation is to find the averaged error between actual and predicted values of a model. RMSE can be calculated using Equation 17.


$$\begin{array}{l} RMSE=\sqrt{\frac{\sum_{i=1}^{n}{\left(predicted_{i}-actual_{i}\right)}^{2}}{n}} \end{array}$$ (17)

It is commonly used to evaluate the performance of regression models.

### 7.6 Cohen's kappa

Cohen's Kappa is mainly used for assessing the level of agreement between two classifiers, and the value falls within the range of −1 to 1. It can be calculated using Equation 18.


$$\begin{array}{l} k=\;\frac{Po-Pe}{1-Pe} \end{array}$$ (18)

It is used in imbalanced datasets and many studies had explicitly reported Cohen's kappa, but it is highly recommended for future studies.

Incorporating metrics like Cohen's Kappa provides a more reliable evaluation of AD classification models, especially in handling imbalanced datasets. DL architectures such as U-Net, Transformer-based models, and Deep Medic outperform traditional models in both accuracy and segmentation quality. Future research should focus on improving segmentation-based hybrid models (CNN and Transformer) for further enhancing AD classification performance.

## 8 Discussion

The discussion is structured to explicitly address the three research questions (RQ1–RQ3) stated in Section 2.1, followed by a broad summary of findings and implications.

### 8.1 Answer to RQ1: what ML/DL techniques have been proposed for AD classification using neuroimaging and clinical data?

Many ML and DL techniques have been applied to AD classification across the 84 reviewed studies. In traditional ML, SVM, Random Forest (RF), K-Nearest Neighbors (KNN), Gradient Boosting/XGBoost, Logistic Regression, and Extreme Learning Machines (ELM) were the most widely applied models on neuroimaging features from MRI, PET, DTI, and EEG. In deep learning, CNNs and their variants (AlexNet, VGG, ResNet, DenseNet, GoogleNet), and Recurrent networks (RNN, LSTM, BiLSTM) were employed for longitudinal and time-series data. Recently, Transformer-based architectures and GANs have emerged for multimodal fusion and synthetic data generation, respectively. Ensemble and hybrid models combining CNNs with classical ML classifiers or Transformers consistently achieved the highest accuracies.

### 8.2 Answer to RQ2: what comparative performance do these models report in terms of accuracy, precision, recall, and F1-score?

Performance varied across model types, dataset size, and classification tasks. Among traditional ML models, accuracy ranged from 62 to 99%, and SVM models consistently achieved 75–95% on ADNI datasets. Among DL models, CNNs achieved 76–99%, ensemble and hybrid approaches achieved the highest values around 98–99%. Though several studies reported high accuracy alongside lower recall, they show class imbalance issues. Multiclass tasks (CN/MCI/AD) consistently achieved lower performance than binary classification (AD vs. CN), highlighting the difficulty of MCI discrimination.

### 8.3 Answer to RQ3: what data modalities and multimodal fusion strategies have demonstrated the highest classification efficacy?

Structural MRI was the most widely used modality, due to its availability and the important volumetric biomarkers (hippocampal atrophy, gray matter density). Multimodal approaches combining MRI with FDG-PET outperformed unimodal MRI across reviewed studies, as PET provides complementary metabolic information not captured by structural imaging alone. Among fusion strategies, pixel-level and voxel-level fusion (GAN-based methods by Zhang et al., 2024; Tu et al., 2024) demonstrated high performance over simple feature concatenation by preserving spatial correspondence between modalities. Knowledge distillation frameworks (Guan et al., 2021) and hierarchical attention-based fusion (Lu et al., 2024) improved multimodal classification, also with less computational cost EEG- based approaches showed low-cost screening but achieved lower accuracy than image based methods. The overall evidence indicates that MRI and PET multimodal fusion with attention-based deep architectures represents high accuracy AD staging, though accessibility, cost, and radiation concerns limit its clinical scalability.

Both ML and DL models have their own advantages and disadvantages. DL models have achieved good results compared to ML models. But still there are some unsolved problems in AD prediction and classification. Each phase of the model like data acquisition, preprocessing, feature selection plays important role in classification accuracy. The above listed model which attained more than 95 percent had trained with small datasets and also had class imbalance problem. Three slices axial, coronal, sagittal has more information (Bi et al., 2020) than single slice data. Multimodal data (Liu et al., 2020) has better performance than the single modal data. At the same time fusing multimodal data is a challenging task (Lin et al., 2021) and has high computational task. Furthermore, simply concatenating or maximizing extracted features from multiple modalities may lead to the loss of important information. GAN is used to resolve (Lu et al., 2024; Zhang et al., 2024; Tu et al., 2024) this problem by fusing information at the voxel level and pixel level. Still more fusion techniques need to be explored. Always using full 4D or 3D images (Ebrahimi et al., 2021) has better performance than the converted images. Some technique (Guan et al., 2021) may help to reduce the cost complexity of multimodal data. In traditional ML approaches, SVM, Random Forest (RF), and kNN have been widely used for AD classification due to their interpretability and lower computational costs. Studies using SVM on ADNI datasets reported classification accuracies between 80 and 90%, but faced challenges in handling high-dimensional neuroimaging data. Feature selection techniques such as Principal Component Analysis (PCA) and Genetic Algorithms (GA) have been employed to improve model efficiency. In DL models, CNNs have shown superior performance in image-based AD classification, with accuracy exceeding 95% on MRI scans. Mousavi et al. (2025) and Zolfaghari et al. (2025) achieved 99 and 99.06%, respectively, with InceptionResNetV2 and dual parallel CNN. The ensemble fusion on MRI datasets highlights the benefits of advanced transfer learning and feature-level fusion for robustness. Athar et al. (2025) showed that targeted data augmentation such as LoG, Prewitt-edge, significantly enhances early AD detection on FDG-PET scans by capturing metabolic changes before structural MRI atrophy appears. Hechkel and Helali (2025) adapted YOLOv11 for multimodal (MRI and DTI) fusion, improving the classification of CN, MCI, and AD. Castellano et al. (2024) achieved better accuracy through 3D MRI and amyloid PET fusion on ADNI. Odusami et al. (2024) combined Simplified Pulse Coupled Neural Network–Laplacian Pyramid fusion with Mobile Vision Transformer and Pareto-optimal quantum optimization on ADNI data for high multiclass classification. Ghassan Al Rahbani et al. (2024) introduced a hybrid ResNet and ADERR post-processing ensemble, reaching up to 99.41% accuracy on public MRI datasets. Sorour et al. (2024) obtained 99.92% with CNN-LSTM on the Kaggle MRI dataset, and Sener et al. (2024) achieved 98–99% with EfficientNetB0 while using McNemar's test to confirm statistically significant model differences. Collectively, these studies underscore the advantages of efficient CNN architectures, multimodal fusion, and advanced augmentation for reliable, high-accuracy AD staging. RNNs and Long Short-Term Memory (LSTM) networks have been applied to longitudinal AD studies, tracking disease progression over time. Transformer-based models have recently outperformed CNNs in feature extraction, but their complexity requires large-scale training datasets. In hybrid and ensemble models, studies combining CNNs with classical ML models (CNN and SVM, CNN and XGBoost) have achieved higher generalizability. Ensemble learning approaches (Bagging, Boosting, and Stacking) have been used to aggregate multiple model predictions for improved robustness. The unique factor about this review is that we analyzed both traditional ML and DL models, including CNNs, Transformers, and Ensemble Learning techniques. Unlike most studies that rely only on MRI, we explored multimodal fusion (MRI, PET, and clinical features) to improve classification accuracy and also focused on segmentation-based techniques for extracting critical brain biomarkers. Instead of relying only on accuracy, we incorporated precision, recall, and Cohen's Kappa analysis to provide a holistic performance evaluation.

## 9 Conclusion and future directions

The task of early AD detection has always been complex. This study provides information on AD-related biomarkers, preprocessing techniques, feature selection, and the use of AI models for AD diagnosis. Most of the preprocessing techniques include removal of skull and unwanted tissues to get brain tissue image with minimal distortion and redundancy. In classification methods SVM, CNN and its variants were frequently used and demonstrated superior performance; however, their effectiveness may be influenced by factors such as small datasets, overlapping training, and testing data. Fusion of data from different modalities (MRI and PET) gives more information than single modality approaches. Despite its advantages, fusion of multimodal is difficult and sometimes it may lead to loss of important information. Advancements such as Voxel level, Pixel level fusion techniques also introduced for the better classification. The best combination of biomarkers and an efficient multimodal fusion approach has to be explored for future research. More advanced preprocessing methods, such as automated feature engineering and DL-based feature extraction, are needed to enhance accuracy. In data augmentation, more sophisticated methods such as GANs could be explored to generate synthetic data and address data imbalance issues. Future studies should report a broader range of metrics, including Cohen's Kappa, AUC-ROC to provide a more comprehensive evaluation of model performance. Cross-modal learning techniques, where models can learn from one modality and apply the knowledge to another, should be explored to enhance the performance of multimodal models. By addressing these challenges, AD diagnosis can become more interpretable, leading to early intervention.
